# Temperature-dependent conformational dynamics govern regioselectivity in a CYP152 decarboxylase

**DOI:** 10.1016/j.jbc.2026.111309

**Published:** 2026-02-26

**Authors:** Mayara C. Avila, Leticia L. Rade, Amanda S. Souza, Ricardo R. de Melo, Celio C. Oliveira, Maria R. de Moraes, Everton E.D. Silva, Wesley C. Generoso, Gabriela F. Persinoti, Carlos H.I. Ramos, Thomas M. Makris, Leticia M. Zanphorlin

**Affiliations:** 1 Brazilian Biorenewables National Laboratory (LNBR), Brazilian Center for Research in Energy and Materials (CNPEM), Campinas, SP, Brazil; 2 Interinstitutional Graduate Program in Bioenergy (USP/UNICAMP/UNESP), Campinas, SP, Brazil; 3 Institute of Chemistry, State University of Campinas, Campinas, SP, Brazil; 4 Department of Molecular and Structural Biochemistry, North Carolina State University, Raleigh, North Carolina, USA

**Keywords:** cytochrome P450, biofuel, decarboxylase, enzyme mechanism, enzyme kinetics, molecular dynamics

## Abstract

Peroxygenases from the CYP152 family catalyze oxidative transformations of fatty acids using hydrogen peroxide and bypass the canonical redox partners. A subset of these enzymes performs fatty acid decarboxylation to generate terminal alkenes, which serve as key intermediates for renewable fuels and petrochemicals. A central unanswered question is how CYP152 enzymes orchestrate the branching between decarboxylation and hydroxylation, a balance that ultimately defines their utility as selective biocatalysts. Here, we uncover a temperature-dependent switch in the peroxygenase from *Nosocomiicoccus massiliensis* (OleT_NS_) that governs its regioselectivity. OleT_NS_ displays a distinctive temperature-dependent catalytic profile, with enhanced β-regioselectivity at milder temperatures, a behavior not previously reported for other members of the family. Our analyses show that its enhanced chemoselectivity arises from coordinated motions between the F-G loop and catalytic His85, which promote deeper substrate burial and selectively favor decarboxylation. At elevated temperatures, this coordination is disrupted, leading to rearranged hydrogen-bonding networks and decreased alkene yields. The combination of loop flexibility and a charged surface typical of cold-active enzymes provides a layer of catalytic control. OleT_NS_ establishes a new model for understanding how structural dynamics and thermodynamic adaptation shape the reactivity of P450 enzymes, highlighting principles for engineering selective biocatalysts for sustainable alkene production.

Cytochrome P450 enzymes constitute a vast and diverse superfamily of monooxygenases characterized by the presence of a heme prosthetic group. These enzymes catalyze the oxidative metabolism of a broad range of substrates and play pivotal roles in areas such as bioremediation, agriculture, and the pharmaceutical industry (1, 2). While most P450 enzymes require redox partners to transfer electrons from NADH or NADPH for catalytic turnover, members of the CYP152 family have evolved a unique catalytic strategy known as the “peroxide shunt” pathway. In this alternative pathway, hydrogen peroxide (H_2_O_2_) functions simultaneously as the oxygen donor and oxidizing agent (3), allowing these enzymes, known as peroxygenases, to function without the need for intricate redox partner systems. The catalytic cycle of these enzymes bypasses the initial electron transfer steps typical of the classical P450 mechanism. Instead, the substrate-bound enzyme reacts directly with H_2_O_2_ to form the high-valent intermediate known as Compound I (4, 5). Notably, in the last years, some CYP152 enzymes have been shown to catalyze an alternative oxidative decarboxylation of fatty acids to yield terminal alkenes and CO_2_ alongside α- and β-hydroxylated products. This reactivity is of particular relevance, as alkenes represent strategic building blocks for the petrochemical industry and as promising renewable precursors for advanced biofuels.

Mechanistic investigations have revealed that both Compounds I and II intermediates participate as key intermediates in alkene formation by P450 decarboxylases. For Compound II to generate the final alkene product, it circumvents radical recombination (*i*.*e*. oxygen rebound) and most likely involves over-oxidation of the substrate to form a carbocationic intermediate. Studies have shown that the chemoselectivity and product distribution are modulated by substrate orientation within the catalytic pocket, suggesting that precise recognition and binding interactions are central to promoting decarboxylation over hydroxylation (6). For instance, OleT_JE_ features an extended F-G loop that contributes to binding selectivity, particularly for long-chain substrates (6). OleTP_RN_ presents a hydrophobic cradle at the distal end of the active site that stabilizes substrate positioning and facilitates product release (7). In contrast, OleTP_CL_ contains an elongated catalytic pocket, and its decarboxylation activity is governed by a conformational rearrangement of a phenylalanine residue located at the end of the catalytic pocket (8).

Despite these advances, a major open question remains in the field: what governs the branching in the chemoselectivity in CYP152 enzymes? Understanding this branching mechanism is critical not only for advancing our knowledge in the field but also for enabling tailored reactivity for biotechnological applications. In a previous study aimed at elucidating patterns underlying the oxidative mechanisms within the CYP152 family, we employed a sequence similarity network (SSN) approach that enabled the clustering of hundreds of enzyme sequences into distinct functional and structural groups (8). This analysis identified isofunctional clusters, with some enriched enzymes exhibiting confirmed decarboxylation activity, whereas others were predominantly associated with hydroxylation-dominant functional profiles (8). A systematic examination, including phylogenetic analysis of the cluster containing the OleT_JE_ decarboxylase, revealed that not only multiple *Jeotgalicoccus* species but also a unique representative from a distinct genus, *Nosocomiicoccus massiliensis*, were gathered on a branch (Fig. S1). This finding is of particular significance, given that the bacterium was originally isolated from the fecal microbiota of an HIV-positive patient presenting with advanced immunodeficiency (9). Although discovered over a decade ago, the enzymatic repertoire of this organism remains poorly characterized. To address this knowledge gap, we investigated its oxidative potential by functionally characterizing the encoded CYP152 peroxygenase. Building on this, here we demonstrate that the predicted peroxygenase from *N*. *massiliensis* (OleT_NS_) exhibits predominant decarboxylation activity, and it can form the high-valent intermediates Compound I and Compound II. Remarkably, OleT_NS_ retains high catalytic efficiency at milder temperatures, displaying a temperature-dependent catalytic profile that is uncommon among CYP152 enzymes. Under these conditions, it shows exclusive β-regiochemistry, markedly enhancing alkene yields compared with the broader regioselectivity observed at higher temperatures. By integrating biochemical assays with structural and dynamic analyses, we elucidate that its enhanced chemoselectivity is linked to correlated motions involving the F-G loop and the catalytic His85, which promote deeper substrate burial and likely favor the decarboxylation pathway. This is particularly interesting since, although the F-G loop is described as a key structural determinant in CYP152 enzymes by anchoring the substrate in a productive orientation and restricting its mobility, our findings reveal a strikingly different role in OleT_NS_ (6). Therefore, these results uncover how temperature-dependent conformational dynamics and catalytic fine-tuning govern alkene formation, providing new mechanistic insights into the structure-function relationships of peroxygenases. Altogether, this work not only expands the enzymatic repertoire of a poorly characterized bacterium but also highlights how thermodynamic properties and structural dynamics decisively modulate the chemoselectivity of P450 peroxygenases.

## Results

### Development of a self-sufficient system for functional OleT_NS_ expression

The efficient heterologous expression of cytochrome P450 enzymes, such as OleT_NS_, in *Escherichia coli* is fundamentally constrained by the restricted intracellular availability of heme, an essential prosthetic group required for proper folding and catalytic activity (10, 11, 12). Conventional approaches to overcome this limitation typically rely on supplementation with 5-aminolevulinic acid (ALA), a key precursor in heme biosynthesis. However, the continuous exogenous supply of ALA required to sustain functional P450 expression represents a high-cost and industrially impractical strategy. To address this metabolic bottleneck, an *E*. *coli* system was designed to redirect bacterial metabolic flux to enhance endogenous heme biosynthesis (Fig. S2). *E*. *coli* natively utilizes the C5 pathway for ALA biosynthesis, which originates from L-glutamate and involves a series of tRNA-dependent enzymatic reactions. In contrast, many eukaryotes and certain bacteria produce ALA through the C4 pathway, by a single-step catalysis involving succinyl-CoA and L-glycine (13). To mitigate the incorporation of ALA into the cultivation media and boost metabolic flux toward heme biosynthesis, the C4 pathway in *E*. *coli* was enabled through overexpression of the hemA_MT_ gene, which encodes the ALA synthase enzyme (Table S1). To this end, the codon-optimized hemA_MT_ gene was integrated into the pG-TF2 plasmid under the glpT promoter and expressed in *E*. *coli*, yielding a red-pigmented recombinant strain, consistent with substantial intracellular heme accumulation. This approach successfully achieved OleT_NS_ expression levels of around 16 mg/L in Terrific Broth medium, establishing a self-sufficient platform for P450 expression that eliminates the need for costly exogenous precursors.

Spectroscopic analysis of purified OleT_NS_ revealed a characteristic Soret band at 423 nm in the UV–visible spectrum, accompanied by the α and β bands at 568 nm and 535 nm, respectively (Fig. S3*A*). To investigate substrate binding, UV–visible difference titrations were performed using fatty acids of varying chain lengths (Fig. S4, Table S2). For shorter-chain substrates (C10, C12, and C14), only modest spectral changes were observed upon substrate addition. In contrast, titration with longer-chain fatty acids resulted in a pronounced shift of the Soret band toward shorter wavelengths. Consistently, analysis of the titration data shows lower apparent dissociation constants (*K*d) for longer-chain fatty acids, indicating higher binding affinity of OleT_NS_ for these substrates.

**Figure 1:**
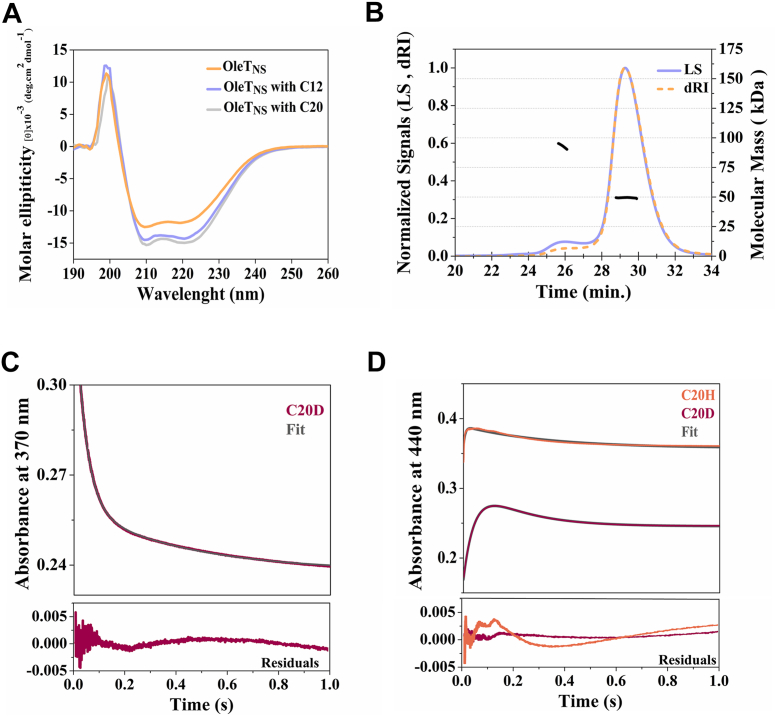
**Structural and kinetic characterization of OleT**_**NS**_. *A*, circular dichroism spectra of OleT_NS_ in its apo form (*orange*), bound to lauric acid (*purple*), and arachidonic acid (*gray*). *B*, molecular mass analysis of OleT_NS_ by size-exclusion chromatography coupled to multi-angle light scattering indicates a predominantly monomeric state with a minor dimeric population. *C*, single-wavelength kinetic traces at 370 nm for C20D, fitted to exponential models, showing the decay of Compound I (residuals displayed below). *D*, absorbance changes at 440 nm for C20H and C20D, reflecting the decay of Compound II; fitted traces and corresponding residuals are shown. The decay rate constants were 24.1 s^-1^ ± 0.6 for Compound I (first phase, measured at 370 nm or 440 nm with C20D) and 183 s^-1^ ± 58 for Compound II (decay phase at 440 nm with C20H). The ratio of the rate constants obtained at 440 nm with C20H and C20D corresponds to a primary kinetic isotope effect of ∼8. Final concentrations after mixing were 10 μM OleT_NS_ and 5 mM arachidonic acid (protonated or deuterated forms) for both the 370 nm and 440 nm measurements.

Complementary circular dichroism (CD) analysis confirmed that OleT_NS_ adopts a mixed α-helical and β-sheet secondary structure consistent with the canonical CYP152 fold (Fig. 1*A*, Table S3). To test conformational resilience, thermal denaturation assays were performed on the apoenzyme and on complexes with lauric (C12) and arachidonic (C20) acids (Fig. S3*B*). The melting temperatures (Tm) remained essentially unchanged at ∼ 51 °C across all conditions, underscoring that substrate binding, irrespective of fatty acid chain length, does not substantially influence the global stability of the protein. The observed Tm closely matches the value previously reported for P450 OleTP_RN_ (52 °C) (7).

Furthermore, size-exclusion chromatography coupled with multi-angle light scattering analysis confirmed that OleT_NS_ exists predominantly as a monomer in solution, with a calculated molecular mass of 48.7 kDa (Fig. 1*B*). This observation is noteworthy in light of recent reports indicating that some members of the CYP152 family can adopt different oligomeric states, including both monomeric and dimeric forms, which may play a functional role in modulating catalytic efficiency and substrate turnover (7, 8).

### OleT_NS_ exhibits temperature-dependent regioselectivity

OleT_NS_ exhibited remarkable tolerance to hydrogen peroxide, maintaining high catalytic efficiency across a broad range of concentrations (0.4–9.0 mM; Fig. S5*A*), consistent with its peroxygenase activity. The enzyme exhibited optimal activity at a near-neutral pH (6.5–7.5; Fig. S5*B*) and was further stimulated by high salt levels (500 mM; Fig. S5*C*), reflecting the halophilic adaptations characteristic of the *Nosocomiicoccus* genus (14).

To elucidate the catalytic mechanism of OleT_NS_, the intermediate Compounds I and II were monitored by stopped-flow experiments using protonated (C20H) or deuterated (C20D) arachidonic acid as the substrate (Fig. 1, *C* and *D*, and Table S4). Decay rate constants were determined by fitting the kinetic traces to two-summed exponential expressions as described in our previous publications (7, 15). Compound I exhibited a rate constant of 24.11 s^-1^ ± 0.60, obtained from the first phase at 370 nm or 440 nm with C20D, with similar values at both wavelengths. The decay of Compound I results in the formation of Compound II, as similarly identified before for OleT_JE_ peroxygenase (16). Photodiode array spectra were also monitored and present the rapid decay of Compound I, followed by the rapid decay of Compound II over 1 s (Fig. S6, *A* and *B*). Compound I was also alternatively monitored at 690 nm (Fig. S6*C* and Table S4). Compound II displayed a rate constant of 183.5 s^-1^ ± 58.0, determined from the decay phase of the 440 nm trace with C20H. The ratio of the rate constants obtained with C20H and C20D at 440 nm corresponds to a primary kinetic isotope effect of ∼ 8, confirming that C-H bond cleavage is the rate-limiting step in Compound I decay, which promotes hydrogen atom transfer from the substrate (6, 15). These observations confirm that OleT_NS_ follows the oxidative cycle found in peroxygenases and monooxygenases from the P450 class (6). Furthermore, Förster resonance energy transfer revealed that rapid mixing of 11-(dansylamino) undecanoic acid Dauda and OleT_NS_ with an excess of H_2_O_2_ revealed that OleT_NS_ undergoes conformational changes on a markedly slower timescale (∼1000 s; Fig. S6*D*) compared to OleT_JE_ (∼30 s), despite showing similar binding constants (k = 0.8 s^-1^ and k = 1.8 s^-1^, respectively) (6). This finding suggests that, while substrate analog binding affinity is conserved, the structural rearrangements required for ligand accommodation and/or product release proceed more slowly in OleT_NS_. Such a delay may indicate differences in the flexibility of regions governing substrate entry and product exit, leading to prolonged conformational relaxation. Although the exact structural origin cannot be assigned solely from Förster resonance energy transfer, the data are consistent with the notion that variations in channel architecture or gating elements contribute to the distinct dynamics of OleT_NS_.

**Figure 2:**
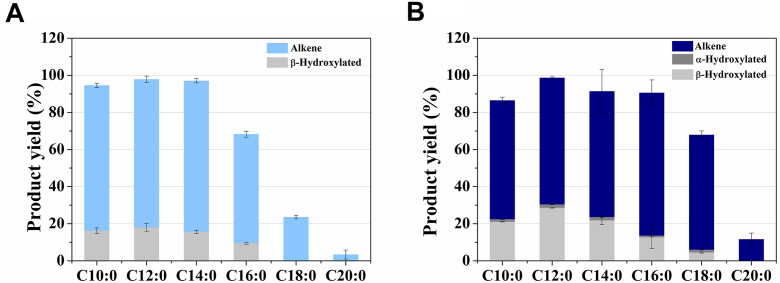
**OleT_NS_ specificity profile at 20 °C and 30 °C**. OleT_NS_ enzymatic activity toward even-chain fatty acids (C10:0–C20:0) at (*A*) 20 °C and (*B*) 30 °C. Reactions were performed using 2 μM enzyme, 500 μM substrate, and 0.8 mM H_2_O_2_ for 1h at 400 rpm.

In terms of temperature dependence, OleT_NS_ exhibited maximal catalytic activity at 20 °C and retained substantial activity at lower temperatures (Fig. S5*D*), distinguishing it from OleTP_RN_ and OleT_JE_, which reach their optimal activity at 35 °C and 32 °C, respectively (7, 17). This unusually catalytic behavior at milder temperatures prompted us to perform turnover reactions with a range of fatty acid substrates at both 20 °C and 30 °C (Fig. 2 and Table S5). At 20 °C, OleT_NS_ displayed strict β-regioselectivity, with no detectable α-hydroxylated products (Fig. 2*A* and Table S5). By contrast, at 30 °C, the enzyme shifted its catalytic profile, generating both α- and β-hydroxylated products along with terminal alkenes for C10-C18 substrates, with C16 showing the highest alkene production (Fig. 2*B* and Table S5). For the C20 substrate, only alkenes were detected, albeit with low conversion, consistent with earlier OleT_JE_ reports (18). Importantly, the yields of alkenes from C10, C12, and C14 substrates were significantly higher at 20 °C than at 30 °C, with decarboxylation/hydroxylation ratios of 4.8, 4.4, and 5.2, respectively (Table S5). These ratios were nearly double those observed at the higher temperature. Taken together, these findings indicate that temperature exerts a direct and previously unreported effect on the regioselectivity and product specificity of OleT_NS_, revealing a new layer of catalytic control within CYP152 enzymes.

### F-G loop as a determinant of regioselectivity in CYP152 decarboxylases

To investigate the determinants of regioselectivity in OleT_NS_, particularly at lower temperatures, we performed mutagenesis targeting the F-G loop, a region known to control substrate orientation in the OleT_JE_ ortholog (6). The AlphaFold-predicted structure of OleT_NS_ reveals a canonical P450 fold and retains the conserved catalytic triad (Phe79, His85, and Arg245) essential for activity and alkene selectivity (18). Its catalytic pocket is highly conserved relative to OleT_JE_, with only minor differences in the substrate-binding region. OleT_NS_ possesses an F-G loop slightly shorter than OleT_JE_ but longer than OleTP_RN_ (Figs. S7 and S8), a decarboxylase from a distinct SSN cluster. In OleT_JE_, the F-G loop anchors the fatty acid substrate, restricting its dynamics and guiding decarboxylation selectivity (6). Conversely, OleTP_RN_, despite a shorter F-G loop, efficiently decarboxylates a broad range of fatty acids, notably including unsaturated ones, aided by a hydrophobic cradle that facilitates product release (7).

**Figure 3:**
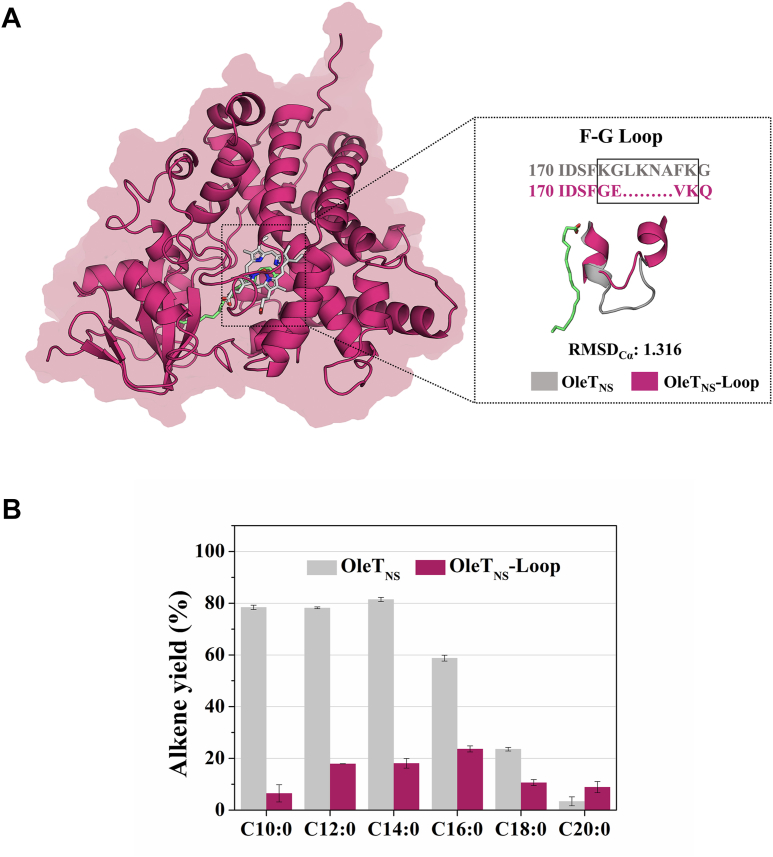
**Effect of F–G Loop mutation on OleT**_**NS**_**catalytic activity**. *A*, overall structure of OleT_NS_ (*pink*), with emphasis on the F-G loop region. The inset shows the native F-G loop sequence (*gray*) compared with the modified sequence introduced in the OleT_NS_-Loop variant (*pink*), resulting in a shortened loop. Structural models of the native and mutant loops are shown in the same colors as their respective sequences. The calculated RMSDcα value between the native and mutant models is 1.316 Å. *B*, comparative activity of native OleT_NS_ and the OleT_NS_-Loop variant toward even-chain fatty acids (C10:0-C20:0). Reactions were performed with 2.0 μM enzyme, 500 μM substrate, and 0.8 mM H_2_O_2_, incubated for 1 h at 20 °C under 400 rpm agitation.

Motivated by these observations, we replaced the F-G loop of OleT_NS_ with the shorter loop from OleTP_RN_, yielding a variant designated OleT_NS_-Loop (Fig. 3*A*). The mutant exhibited proper folding and typical low-spin heme spectra (Fig. S9*A*). Interestingly, OleT_NS_-Loop displayed increased thermal stability relative to the wild-type enzyme, with a ∼ 3 °C increase in melting temperature (Fig. S9*B*). However, fatty acid binding slightly reduced stability, suggesting impaired substrate interactions (Fig. S9*B*). In contrast to wild-type OleT_NS_, OleT_NS_-Loop shows no detectable Soret shift for any tested substrate (Fig. S10). This observation indicates that the loop swap alters substrate binding and/or positioning within the active site. For OleT_NS_-Loop, the absence of a clear spectral response prevented the generation of a quantitative binding curve, and therefore reliable *K*d values could not be determined. Additionally, to assess whether Triton binds to the enzyme, titrations with the wild-type OleT_NS_ and the OleT_NS_-Loop mutant were performed, and no detectable Soret shift was observed for either protein (Fig. S11).

Indeed, the catalytic assays with a broad panel of fatty acids revealed that OleT_NS_-Loop had substantially reduced activity compared to native OleT_NS_ and did not improve efficiency toward unsaturated substrates at either 20 °C or 30 °C (Figs. 3*B* and S12). The F–G loop has been implicated as a selectivity-modulating element in OleT_JE_, where loop mutations affect product profiles. Our OleT_NS_-Loop results extend this concept, showing that alterations in this region directly influence regio- and chemoselectivity, likely by reshaping substrate access and positioning (6). As OleT_NS_ and OleT_JE_ share the same SSN cluster (Fig. S1), while OleTP_RN_ does not, these results suggest that functional divergence within the CYP152 family may, in part, reflect evolutionary adaptation of F-G loop regions. Given the similar temperature-dependent behavior of the mutant, the current data do not clarify whether the F-G loop influences thermodynamic determinants of the OleT_NS_ activity.

### Coupled movements of catalytic histidine and the F-G loop control regioselectivity in OleT_NS_ at milder temperatures

Since the catalytic site and the substrate-binding pocket are highly conserved between OleT_NS_ and OleT_JE_, we then evaluated the peripheral regions of the proteins. Supporting this view, extensive studies on different cold-active enzymes indicate that, although the catalytic residues are generally conserved relative to their thermophilic orthologs, significant differences emerge at the protein surface (19). These differences typically manifest as an enrichment in charged residues, particularly negative ones, which enhanced conformational flexibility and maintained catalytic efficiency below the melting temperature (20). Consistent with this, our comparative analysis revealed that OleT_NS_ contains a higher number of charged residues than OleT_JE_ and OleTP_RN_, many of which are surface-exposed (Figs. S13, S14, and S15). Although OleT_JE_ is already relatively polar, OleT_NS_ is even more so, with an additional two negative and five positively charged residues distributed across its structure. These differences are particularly evident at the protein surface, reinforcing the notion that OleT_NS_ has evolved molecular features that support catalytic activity under milder conditions.

To gain deeper insight into the flexibility of OleT_NS_, we performed molecular dynamics simulations combined with principal component analysis (PCA), allowing us to capture the relevant motions along the trajectories at different temperatures and directly compared them with OleT_JE_. PCA across all conditions revealed that approximately 40% of the variance is explained by the first 5 PCs, ∼ 70% by the first 10 PCs, and ∼ 90% by the first 20 PCs (Fig. S16). Clustering analysis based on the first 20 PCs was then performed and revealed a distinct pattern in which OleT_JE_ at 30 °C and OleT_NS_ at 20 °C displayed elongated, partially overlapping clusters, indicating frequent interconversions between them. In contrast, OleT_JE_ at 20 °C and OleT_NS_ at 30 °C formed well-separated, compact clusters, consistent with more rigid structures. The higher conformational flexibility captured by MD for OleT_NS_ at lower temperatures may point to a mechanistic link between enhanced mobility and catalytic efficiency.

**Figure 4:**
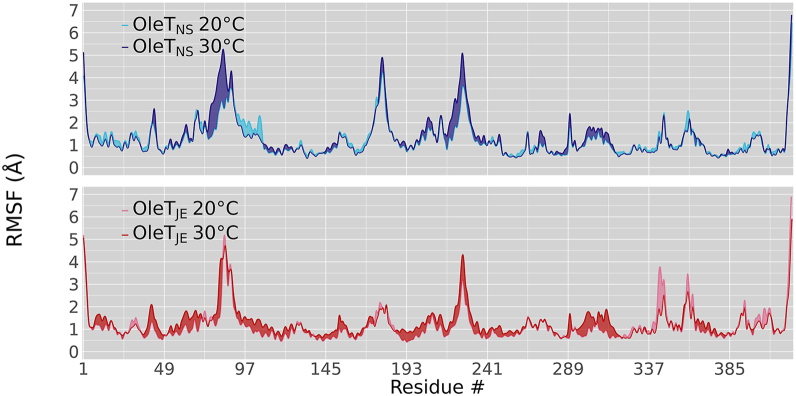
**Conformational dynamics of OleT enzymesupon temperature**. OleT_NS_ (*blue*) and OleT_JE_ (*red*) show a distinct dynamic profile under the same conditions. Lighter shades correspond to simulations at 20 °C, and darker shades to 30 °C. Root mean square fluctuation values of Cα atoms were obtained from principal component analysis-derived pseudotrajectories, constructed from eight independent 200-ns simulations (totaling 1.6 μs per condition).

By analyzing temperature-dependent root mean square fluctuation variation within each enzyme, we observed notable differences, mostly in regions surrounding the F-G loop and the catalytic histidine (His85), both of which are recognized as critical determinants of decarboxylation activity (Fig. 4). In line with the PCA-derived temperature factor analysis (Fig. S17), OleT_NS_ exhibited pronounced motion in its F–G loop, located between amino acids 176 and 181. This finding is surprising because, in the ortholog OleT_JE_, the F-G loop anchors the substrate in the active site, simultaneously restricting the motions while ensuring their productive positioning for efficient decarboxylation (6). Indeed, our MD results show that the F-G loop of OleT_JE_ remains relatively rigid, consistent with its established role in stabilizing the substrate during catalysis (Fig. 4).

**Figure 5:**
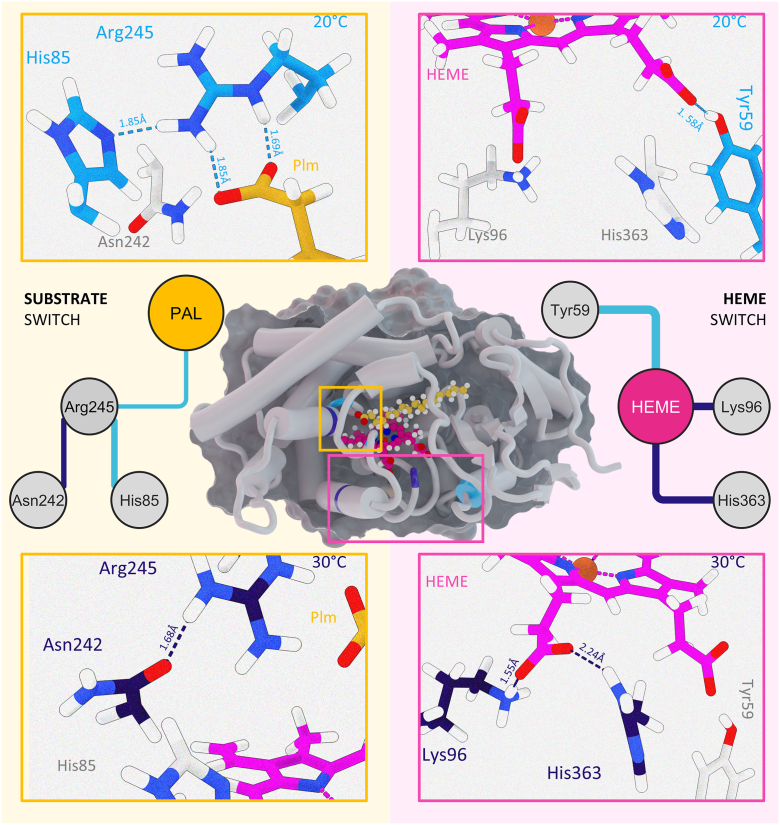
**Temperature-induced switch network coordination in OleT_NS_**. Principal component analysis-derived motions are mapped onto the structural model of OleT_NS_ bound to heme (*magenta*) and the C16 substrate palmitic acid (*yellow*). Insets highlight hydrogen-bond interactions surrounding the substrate (*left*) and the heme group (*right*). Contacts more frequent at 20 °C are shown in *light blue* (*top panels*), whereas those enriched at 30 °C are shown in *dark blue* (*bottom panels*). Distances (Å) and interacting residues are indicated.

These results prompted us to further investigate the interaction patterns in both decarboxylases at different temperatures by performing a detailed analysis of hydrogen bonding along the MD pseudo-trajectories. The data unveiled a distinct temperature-dependent pattern in OleT_NS_. For instance, interactions between Ser101-Glu219 and Asp398-Ser401 were favored at 30 °C but not at lower temperatures (Fig. S18). Interestingly, this analysis revealed that OleT_NS_ undergoes a conformational switch driven by temperature, involving residues directly related to substrate recognition, particularly around the catalytic histidine and the heme prosthetic group. At lower temperatures, the catalytic residue R245 predominantly interacts with both palmitic acid and His85, whereas at higher temperatures it shifts to favor interaction with Asp242 (Fig. 5 and Fig. S19). Similarly, under milder conditions, the heme group is mainly coordinated by Tyr59, a residue previously identified as critical for P450 functionality (21), while at elevated temperatures it interacts mostly with Lys96 and His363 (Figs. 5 and S19). These observations suggest that changes in temperature alter the hydrogen-bonding network and the heme coordination environment, ultimately affecting substrate positioning and catalytic efficiency.

**Figure 6:**
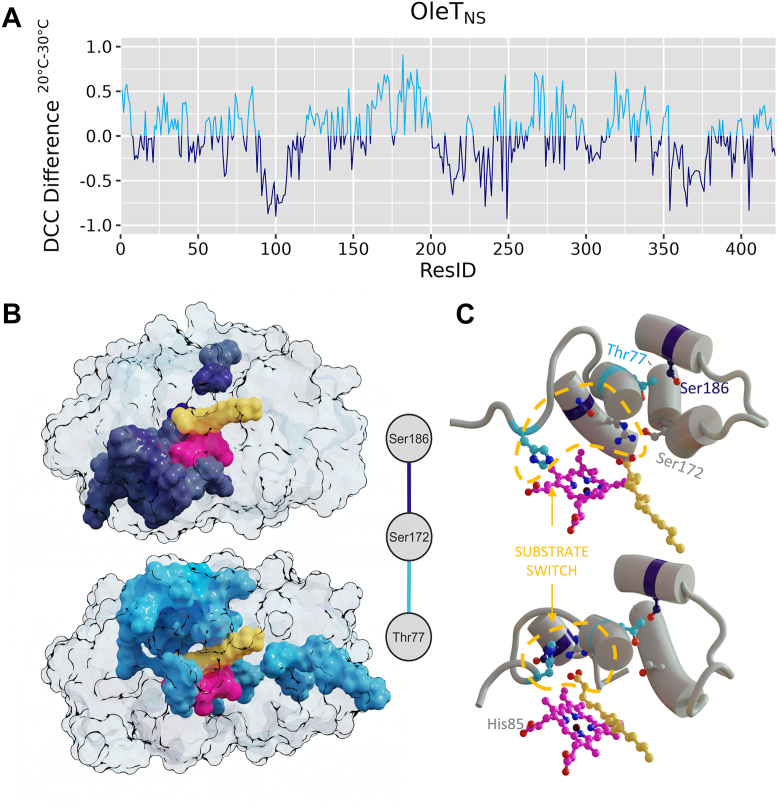
**Temperature effects on OleT_NS_ dynamic cross-correlation**. The z-score metric combines two structural descriptors: (i) the distance between the heme iron and the substrate Cβ atom, and (ii) the Cα-Cβ-heme iron angle. *A*, per-residue variations in the z-score along the OleT_NS_ sequence highlight regions undergoing significant temperature-dependent dynamic shifts. *B*, structural mapping of residues correlated with the z-score at 20 °C and 30 °C, with the heme group in *pink* and the C16 substrate palmitic acid in *yellow*. *C*, hydrogen-bond networks preferentially stabilized at 20 °C are depicted in *light blue* (*top panels*), whereas those enriched at 30 °C are shown in *dark blue* (*bottom panels*).

To further validate temperature-dependent effects on substrate interaction in OleT_NS_, we performed a correlation analysis using a z-score that integrates both the distance between the heme iron and the substrate’s Cβ atom and the angle defined by Cα-Cβ-heme iron, thus capturing substrate proximity and orientation within the active site. This evaluation revealed that movements in the region surrounding the F-G loop in OleT_NS_ are strongly coupled to substrate motions at lower temperatures. Supporting this observation, our hydrogen-bond analysis uncovered temperature-driven conformational changes involving Ser172, a residue located near the F-G loop. At 20 °C, Ser172 predominantly interacts with Thr77, whereas at higher temperatures it preferentially engages Ser186 (Fig. 6 and Fig. S19). Remarkably, this rearrangement is closely coupled to the movement of His85 described above. At elevated temperatures, the Ser172-Ser186 interaction positions the substrate farther from the heme and displaces His85 away from Arg245, which instead establishes interaction with Asp242 (Figs. 6 and S19). In contrast, at lower temperatures, the Ser172-Thr77 interaction maintains His85 coordinated by Arg245, effectively driving the substrate deeper into the catalytic pocket and thereby favoring catalysis (Movie S1).

Such coordinated dynamics, alongside the distinctive surface polarity of OleT_NS_, likely promote productive substrate accommodation and enzyme stabilization at lower temperatures, leading to enhanced alkene formation and dictating regioselectivity. The F-G loop, already recognized as critical in OleT_JE_, also plays a pivotal role in OleT_NS_, but in a different manner, with its motion closely linked to His85 orientation. Both conformational and structural dynamics may contribute to the enhanced chemoselectivity of OleT_NS_ for alkene production at reduced temperatures, highlighting its potential as a promising P450 peroxygenase.

## Discussion

The orientation of the fatty acid within the catalytic pocket of CYP152 decarboxylases plays a central role in determining the reaction outcome, which is bifurcated into decarboxylation and hydroxylation. Additionally, the enzyme's ability to control proton delivery and stabilize transient intermediates can shift the balance toward either hydrogen atom abstraction (leading to hydroxylation) or decarboxylation *via* C-C bond cleavage. Computational simulations have shown that second-sphere coordination interactions can modulate chemoselectivity, with the reaction pathways from Cα and Cβ positions being energetically close (22). In addition to that, DFT calculations have demonstrated that perturbations from external electric fields can alter the order of hydrogen abstraction, thereby modulating selectivity (23, 24). This is particularly intriguing because such electrostatic effects may arise from dipole moments within the highly polarized decarboxylases. For over a decade, OleT_JE_ has served as the primary structural model for investigating determinants of alkene production in CYP152 enzymes. Recently, this knowledge was significantly expanded through the determination of three additional decarboxylase crystal structures, providing deeper insight into the structural mechanisms that govern specificity within the family (7, 8).

While substantial progress has been made in understanding CYP152 catalysis, the structural determinants underlying shifts in chemoselectivity remain only partially resolved. Despite its high sequence similarity to OleT_JE_ and conservation of key catalytic elements, OleT_NS_ exhibits distinctive electrostatic and flexibility patterns consistent with its low-temperature–optimized behavior. Under low-temperature conditions, OleT_NS_ catalyzes oxidation exclusively from the Cβ position, yielding a higher proportion of alkene products. For the first time, our results uncover correlated motions between the F-G loop and the catalytic His85 that enable productive substrate positioning and favor decarboxylation, thereby providing a structural and mechanistic rationale for the enhanced chemoselectivity. In contrast to OleT_JE_, where the F-G loop restricts substrate dynamics, OleT_NS_ relies on coordinated loop rearrangements to guide catalysis, while its highly charged protein surface contributes to structural stability at low temperatures. Altogether, this work expands the mechanistic framework for peroxygenase chemoselectivity and highlights OleT_NS_ as a model system for understanding how structural dynamics and temperature-dependent effects interplay to shape productive P450 catalysis.

## Experimental procedures

### Phylogenetic analysis

The 60 protein sequences from the OleT_JE_ cluster previously reported by Generoso *et al*. (8) were aligned using MAFFT (25), and alignment columns containing more than 70% gaps were excluded. The filtered dataset was analyzed with IQ-TREE (26), where the LG + I + G4 substitution model comprising the LG matrix, a proportion of invariable sites (I), and gamma-distributed rate heterogeneity across four categories (G4), was selected under the Bayesian Information Criterion. A consensus phylogeny was generated from 100 bootstrap replicates. For the subclade containing OleT_JE_ and OleT_NS_, the 10 sequences were modeled in AlphaFold3 with a heme B prosthetic group and a palmitic acid molecule (27). The top-ranked models were subsequently aligned by structure using the FoldMason method (28), and the resulting structure-based sequence alignment was analyzed with IQ-TREE 2. In this case, the LG + G4 model was selected by Bayesian Information Criterion, and a consensus tree was generated from 100 bootstrap replicates. Visualization of phylogenetic trees was performed using the iTOL web server (29).

### Construction of a heme biosynthesis platform in *E*. *coli*

The pG-TF2 plasmid (Takara Bio) was selected as the cloning backbone for the 5-aminolevulinate synthase (ALAS; named hemA_MT_) gene. This vector system was selected due to its constitutive co-expression of the chaperone ensemble GroEL/GroES and trigger factor (Tig), which synergistically enhances the folding and solubility of cytochrome P450s. The hemA_MT_ coding sequence (Table S1), previously identified through metagenomic mining of the LNBR database, was codon-optimized for *E*. *coli* and synthesized by GenScript. The gene was placed under the transcriptional control of the glpT promoter (BBa_J72163) and flanked downstream by the rrnB terminator. Cloning was performed using Gibson Assembly. Positive clones were subjected to plasmid extraction, and the integrity of the construction was verified by Sanger sequencing, confirming the correct assembly of the promoter, coding sequence, and terminator junctions. The final construct, designated pG-TF2-H4, harbors both the hemA_MT_ gene and the chaperone expression system.

### Heterologous expression and purification of OleTs and mutants

The OleT_NS_ and mutant, cloned into the pET28a vector, were co-transformed with the pG-TF2-H4 plasmid into *E*. *coli* BL21(DE3). The OleT_NS_ gene was codon-optimized for *E*. *coli* and synthesized by GenScript. Briefly, the transformed strains were cultured in 500 ml of selective Terrific Broth medium supplemented with 40 μg/ml chloramphenicol, 50 μg/ml kanamycin, and 125 mg/L thiamine hydrochloride, at 37 °C. Once the optical density (OD_600nm_) of the cultures reached 0.8, the growth temperature was lowered to 18 °C. Subsequently, 500 μM isopropyl-β-D-thiogalactopyranoside, 5 μM FeCl_3,_ and 10 μg/ml tetracycline were added. The cultures were incubated at 18 °C for 16 h. Cells were harvested by centrifugation at 7000 x g for 20 min at 4 °C and resuspended in Buffer A (30 mM potassium phosphate, pH 7.5, 300 mM NaCl, 5% glycerol, and 20 mM imidazole). Cell disruption was carried out by sonication using a Vibracell device (Sonics and Materials). Cell lysis was carried out by 1 min of sonication (40% amplitude, no pauses), followed by 5 min stirring at 4 °C, repeated five times. The cell lysate was centrifuged at 16,000 x g for 50 min, and the clear supernatant was loaded onto a 5 ml His-Trap chelating HP column (GE Healthcare Biosciences), which had been pre-equilibrated with Buffer A. The target protein was eluted using a gradient of imidazole (20–300 mM) in Buffer A. The eluted sample was then loaded onto a HiLoad 16/600 Superdex 200 pg column, equilibrated with 30 mM potassium phosphate, pH 7.5, 150 mM NaCl, and 5% glycerol. The heterologous expression and enzyme purification were analyzed by sodium dodecyl sulfate polyacrylamide gel electrophoresis (SDS-PAGE, 12%).

### Titration of fatty acid binders and monitoring by UV-visible

In the resting state, the heme group of CYP152 enzymes is coordinated by a thiolated cysteine and a water molecule, producing a characteristic Low-Spin (LS) absorbance peak at 423 nm. Substrate binding induces a spin-state shift of the heme iron to High-Spin (HS), shifting the absorbance peak to 390 to 395 nm (30). This spectral change allows the quantification of the LS-to-HS transition and the determination of the dissociation constant (*K*d) for each substrate. Fatty acid binding to OleT_NS_ was evaluated using even-chain fatty acids from C10 to C20 (Sigma Aldrich), prepared in ethanol:Triton X-100 (70:30 v/v). Titrations were carried out by gradual addition of substrate to 1 ml of 5 μM enzyme in buffer (100 mM potassium phosphate, pH 7.5, 500 mM NaCl, 5% glycerol). UV-vis spectra (200–800 nm) were recorded after each addition. The absorbance change (ΔA) between LS (423 nm) and HS (395 nm) maxima was plotted against substrate concentration, and *K*d values were obtained by fitting the data to Morrison’s quadratic equation.

### Secondary structure and thermal stability assessment by circular dichroism (CD)

Far-UV CD spectra of OleT_NS_ and its mutant were recorded using a JASCO J-815 CD spectrometer with a CDF-426S Peltier temperature control system. Approximately 3 μM of each enzyme in buffer (100 mM potassium phosphate, pH 7.5, 500 mM NaCl, 5% glycerol) was used at 20 °C in a 1 mm path-length quartz cuvette. Spectra represent the average of ten scans, measured from 190 to 260 nm with 2 nm bandwidth and 4 s response time. For thermal unfolding, ellipticity at 208, 218, and 222 nm was monitored while increasing temperature from 20 to 100 °C at 1 °C min^-1^. Observed ellipticity (θ) was normalized by protein concentration and reported as mean residue molar ellipticity (θ degree^-1^ cm^2^ dmol^-1^). Temperatures at the middle of the transition (Tm) were determined by Boltzmann sigmoidal fitting of the denaturation curves. Data analysis and plotting were performed using Origin 8.1 (OriginLab). Secondary structure deconvolution of the collected CD data for the OleTNS enzyme was performed using the BeStSel algorithm (31). The analysis was applied to baseline-corrected spectra expressed in mean residue ellipticity units, and the percentages of α-helix, β-strand (parallel/antiparallel), turn, and unordered structures were obtained from the server output.

### Analytical size-exclusion chromatography coupled to multi-angle light scattering

The oligomeric state of OleT_NS_ in solution was determined by size-exclusion chromatography coupled to multi-angle light scattering. Samples (100 μl, 2 mg/ml) were injected onto a Superdex 200 10/300 column (Cytiva) and separated using an Agilent 1260 Infinity II HPLC system with an isocratic pump and autosampler (Agilent Technology). The mobile phase consisted of 100 mM potassium phosphate (pH 7.5), 150 mM NaCl, and 5% glycerol, at a flow rate of 0.5 ml/min. Protein elution was monitored using an 8-angle static light scattering detector (DAWN 8) coupled to an Optilab refractive index detector (Wyatt Technology). The column was calibrated with bovine serum albumin as a molecular weight standard, and data were analyzed using ASTRA 8.1.2 software (Wyatt Technology; https://www.wyatt.com/products/software/astra-8-launch.html).

### Ferrous oxidation xylenol-orange (FOX) assays

To determine the optimal NaCl concentration and pH for OleT_NS_ activity, ferrous oxidation xylenol-orange (FOX) assays were performed (32). These assays detect peroxide consumption in the reaction. Reactions were conducted in 96-well plates (100 μl final volume) containing 1 μM OleT_NS_ and 200 μM myristic acid in buffer (100 mM potassium phosphate, pH 7.5, 100 mM NaCl, 5% glycerol). Reactions were incubated at 30 °C for 5 min, followed by the addition of 100 μM hydrogen peroxide and further incubation at 30 °C for 10 min (Veriti Thermal Cycler, Applied Biosystems). Reactions were quenched by heating at 70 °C for 5 min. Then, 25 μl of each sample was mixed with 75 μl ultrapure water and 100 μl of FOX solution (1 ml 10X FOX stock, 300 μl ethanol, 8.7 ml water). The 10X FOX stock contained 2.5 mM xylenol orange, 2.5 mM FeSO_4_, and 0.5 M H_2_SO_4_. Samples were incubated at 60 °C for 5 min, and absorbance was measured at 560 nm using an Infinite 200 PRO microplate reader (TECAN). Negative controls without enzymes were included. pH dependence was tested from 4 to 10 using citrate (pH 4.0–5.5), phosphate (pH 6.0–8.0), Tris-HCl (pH 9.0), and glycine (pH 10.0). NaCl tolerance was assessed from 0 to 1 M. Relative activity (%) was calculated based on the maximal catalytic activity under optimal conditions. All experiments were performed in triplicate.

### Fatty acid substrate conversion assays

Activity assays were performed in 500 μl reaction volumes containing 2 μM enzyme, 500 μM fatty acid (C10-C20), and 600 μM H_2_O_2_. Hydrogen peroxide was added to positive controls in six incremental steps of 1 μl every 10 min over 1 h. Negative controls without enzyme were included. Reactions were incubated at 20 °C or 30 °C for 60 min with shaking at 750 rpm using an Eppendorf Thermomixer. The reaction was immediately stopped with 5 μl of 50% HCl. The reaction products were derivatized and analyzed by gas chromatography (7890A, Agilent Technologies) using an RTx-5MS column (30 m × 0.25 mm × 0.25 μm) under previously established conditions (7, 8). All assays were conducted in triplicate.

### Transient kinetics measurements and analysis

OleT_NS_ was pretreated with a 10-fold molar excess of hydrogen peroxide to remove any adventitiously bound fatty acids. Treated samples were desalted using a PD-10 column (GE Healthcare) to eliminate small molecules. Enzyme-substrate complexes were prepared by mixing 20 μM OleT_NS_ with 100 μM fatty acid and incubating on ice for 30 min. Methods for kinetic measurements and data analysis were performed as previously described (6, 16).

### Site-directed mutagenesis

The OleT_NS_ mutant, designated OleT_NS_-Loop, was generated by inverse PCR using the primer pair GACATGGATATCA (forward) and CATTCGGTGAAG (reverse). Primers were designed with complementary sequences of 15 nt and melting temperatures of 50 °C, incorporating the desired codon changes. PCR amplicons were circularized *via* Gibson assembly. The mutation was confirmed by gel electrophoresis and Sanger sequencing at the High-Performance Sequencing Laboratory (LNBR/CNPEM).

### Structural analysis

The predicted structure of OleT_NS_ was generated using AlphaFold3(27). Molecular visualization and surface analysis of OleT_NS_, OleTP_RN_ (PDB: 8D8P), and OleT_JE_ (PDB: 4L40) were performed using PyMOL (33). To standardize the analysis, specific PyMOL scripts were applied. The “*FindSurfaceResidues*” script was used to identify and color surface residues, enabling the quantification of hydrophobic and hydrophilic residues. Subsequently, the “*FindSurfaceCharged*” script was employed to identify charged residues, including arginine (R), aspartic acid (D), glutamic acid (E), histidine (H), and lysine (K).

### Molecular dynamics simulations and analysis

OleT_JE_ and OleT_NS_ structures were modeled with the heme B cofactor and C16 substrates using AlphaFold3 (27). Systems were prepared in CHARMM-GUI with the CHARMM36 force field at pH 7.0 (34). The force field was manually modified to include parameters for the Cys-Fe coordination bond, with a restraint ensuring that the Fe and S atoms remained within 0.25 nm (8). Histidine protonation states were assigned with PropKa (35). In OleT_JE_, residues 92, 259, 325, and 363 were set to HSE, while 85, 120, 210, and 222 were HSD. In OleT_NS_, residue 92 was HSE, and 85, 136, 222, 264, 305, 325, and 363 were HSD. Systems were solved with TIP3P water molecules, and ionic strength was neutralized to 150 mM NaCl using Monte Carlo ion placement. All systems were minimized and equilibrated in the NPT ensemble, with temperature maintained by a V-rescale thermostat and pressure controlled by a C-rescale barostat with semi-isotropic coupling at either 293.15 K or 303.15 K. Molecular dynamics simulations of 200 ns were run in GROMACS 2022 (36) with a 2-femtosecond timestep, using at least eight independent replicates per system.

Trajectory frames were concatenated and aligned to the protein backbone heavy atoms before PCA. PCA was performed on all heavy-atom coordinates until 75% of cumulative variance was reached. A per residue essential dynamics score, B_i_^PCA^, was computed as a composite temperature factor like metric by summing across all retained principal components the squared atomic loadings weighted by the corresponding eigenvalues and scaling by 8π^2^/3 (37). Atomic scores were averaged within each residue to obtain an absolute per residue profile. Clustering in the reduced PC space was carried out with HDBSCAN using the Manhattan metric and leaf selection method (38). Medoid frames were identified as the structures minimizing the average pairwise RMSD and saved as representative states.

Pairwise RMSDs among medoids were then calculated in VMD (39) using selections that included the protein backbone (residues 11–412), heme, and the palmitate substrate, excluding hydrogens. A nearest-neighbor traversal of the RMSD matrix was used to generate an ordered sequence of states, which was assembled into a reordered pseudotrajectory with alignment applied between consecutive frames to reduce drift. To capture transitions, linear morph interpolation was performed between successive medoids, with the number of interpolated frames scaled to the RMSD between them. These reordered and interpolated pseudotrajectories were then used for root mean square fluctuation calculations and visualization of conformational dynamics. Hydrogen bonds were identified in centroid frames with a distance cutoff of 3.0 Å and an angle cutoff of 20°, and occupancies were quantified using the VMD HBonds plugin version 1.2. Differential hydrogen-bond networks between 20 °C and 30 °C were analyzed with Cytoscape v3.10 (40). PCA variance plots and clustered trajectory scatterplots were generated and structural and interaction visualizations were produced in UCSF ChimeraX (41) and Blender version 4.3.

## Data availability

The data supporting this article have been included as part of the supporting information.

## Supporting information

This article contains supporting information (7, 31).

## Conflict of interest

The authors declare that they have no conflicts of interest with the contents of this article.
