# Supplementary material for: Temperature-dependent conformational dynamics govern regioselectivity in a CYP152 decarboxylase
Source: J Biol Chem. 2026 Feb 26;302(4):111309. doi: 10.1016/j.jbc.2026.111309 (PMC13049924; doi:10.1016/j.jbc.2026.111309)
Supplement: Supplementary Material — 1 [file mmc1.pdf]

Movie S1. $OleT_{NS}$ Catalytic Mechanism with palmitic acid (C16:0, yellow) at 20 °C shows increased hydrogen bond frequencies between residue pairs Arg245–His85 and Ser172–Thr77 (light blue), which help stabilize the substrate in the catalytic pocket by keeping it in a closed conformation, aided by the F-G loop movement that pushes the substrate toward the active site. At 30 °C, residue pairs Arg245–Asn242 and Ser172–Ser186 (dark blue) form stronger interactions, resulting in a more open catalytic pocket and less conformational constraint for substrate positioning. The heme B group is shown in hot pink.
