# Supplementary material for: Temperature-dependent conformational dynamics govern regioselectivity in a CYP152 decarboxylase
Source: J Biol Chem. 2026 Feb 26;302(4):111309. doi: 10.1016/j.jbc.2026.111309 (PMC13049924; doi:10.1016/j.jbc.2026.111309)
Supplement: Supplementary Material 3 [file mmc3.pdf]

SUPPLEMENTAL INFORMATION

Temperature-dependent conformational dynamics govern regioselectivity in a CYP152 decarboxylase

Mayara C. Avila[1,2], Leticia L. Rade[1], Amanda S. Souza[1], Ricardo R. de Melo[1], Celio C. Oliveira[1], Maria R. de Moraes[1], Everton E. D. Silva[1], Wesley C. Generoso[1], Gabriela F. Persinoti[1], Carlos H. I. Ramos[3], Thomas M. Makris[4], Leticia M. Zanphorlin[1*]

[1] Brazilian Biorenewables National Laboratory (LNBR), Brazilian Center for Research in Energy and Materials (CNPEM), Campinas, SP, Brazil.

[2] Interinstitutional Graduate Program in Bioenergy (USP/UNICAMP/UNESP), 330 Cora Coralina Street, Cidade Universitária, Campinas, SP, Brazil.

[3] Institute of Chemistry, State University of Campinas, Campinas, SP, Brazil.

[4] Department of Molecular and Structural Biochemistry, North Carolina State University, Raleigh, North Carolina, United States.

*Corresponding author: leticia.zanphorlin@lnbr.cnpem.br

Table S1. Amino acid sequences of proteins investigated in this study.

Name	Protein sequence
$OleT_{NS}$<br>(Peroxygenase from *Nosocomiicoccus massiliensis*)	MAKIKRDKGLDNTLKVFKQGYLYTTNQRERLGAEVFETRALGGK<br>SYVVLSGKEGAELFYDNDKIEREGGLPKRVVNTLFGKGAIHTTTG<br>KQHIDRKALFMSLMTEGNLEYVRKLTRNYWKANTARMEAMGDV<br>NVYHESIVLLTRIGMRWAGVTAPEEEIERIAEDMDIMIDSFKGLGNA<br>FKGYKSSKDARKRVEDWLEDQIIKTRNGEINPPKGSSLYELAHWRD<br>YEGNQMDSRLAGIDLMNTFRPLIAINRFVSYGVLALYEHPEAIRKM<br>STVEDYPYMFAQEVRRFYPFVPFLPGKTKVEVEHKGVTIPKDQRLV<br>IDVYGTLHSEELWDQPNKFIPERFKDWDGSPFDMIPQGGGDYWTN<br>HRCAGEWITIIIMEETMKYFANEITWDVPEQDLTIDLNSIPGYINSG<br>MVINNVREKVDRF
$hemA_{mt}$<br>(5-Aminolevulinate Synthase - ALAS)	MDYETFFRTELDGLRREGRYRVFADLERQAGRFPRATYHGEGGPR<br>EITVWCSNDYLGMGQHRAVLAAMHEALDSCGAGAGGTRNIGGTN<br>HYHVLLEQELADLHGKEAALLFNSGYMSNWASLGTLAARIPGCV<br>VLTDALNHASMIEGIRHSRAERQIFAHNDPDDLRRKLAALDPARPK<br>LIAFESVYSMDGDIAPIETFCDIAEEFGAMTYIDEVHAVGLYGPRGG<br>GVSERDGLSHRLTVIEGTLAKAFGVMGGYIAGSAAMCDFVRSFAS<br>GFIFSSSLPPPVAAGALAAIRHLKTSTIERERHQDRVATLRSRLDAAG<br>LPHLANPSHIVPVMVGDPVLCKAVSDELLERFDIYVQPINYPTVPR<br>GTERLRITPSPLHSDADIDHLVEGLSAIWGRVGLQRAA

Table S2. *K*d values for $OleT_{NS}$ binding to different fatty acids and the corresponding percentage of Low-Spin to High-Spin transition (LS→HS %).

**Fatty acid**	***K*d (µM)**	
	**$OleT_{NS}$**	**HS%**
**C10**	38.34 ± 29	97.8
**C12**	75.45 ± 36	80.8
**C14**	36.48 ± 3	91.2
**C16**	11.81 ± 2.95	92.2
**C18**	1.58 ± 0.14	94.1
**C18:1**	6.25 ± 3.35	66.7
**C20**	1.65 ± 0.55	54.1

Table S3. CD-derived secondary structure content of $OleT_{NS}$ in the absence (apo) and presence of C12 fatty acid, obtained by BeStSel deconvolution (1). For comparison purposes, CD data of the $OleTP_{RN}$ enzyme (PDB: 8D8P) were also retrieved and deconvoluted using the same approach (2).

CYP152 enzymes	***Helix (%)***	***β-sheet (%)***	***Coil/Other (%)***
**$OleT_{NS}$ (apo)**	26	20	43
**$OleT_{NS}$ + C12**	29	22	39
**$OleTP_{RN}$**	50	10	40

Table S4. Rate constants and amplitude obtained with stopped-flow spectroscopy of the $OleT_{NS}$: Arachidonic acid (deuterated or protonated) complex and excess of hydrogen peroxide at 4 ºC.

	**370 nm**				**440 nm**				**690 nm**	
**$OleT_{NS}$ with C20D**	A_1	K_1	A_2	K_2	A_1	K_1	A_2	K_2	A_1	K_1
	0.06 ± 0.004	24.70 ± 2.390	0.02 ± 0.001	2.10 ± 0.900	- 0.10 ± 0.010	23.50 ± 0.300	0.07 ± 0.001	5.40 ± 0.100	0.01 ± 0.001	25.10 ± 1.470
**$OleT_{NS}$ with C20H**	A_1	K_1	A_2	K_2	A_1	K_1	A_2	K_2	**-**	**-**
	0.02 ± 0.002	319.00 ± 7.000	0.04 ± 0.020	0.10 ± 0.010	- 0.04 ± 0.001	183.50 ± 58.000	-0.02 ± 0.001	2.00 ± 0.760	-	-

Table S5. Product distribution and decarboxylation/hydroxylation ratios of $OleT_{NS}$ with even-chain fatty acids (C10:0-C20:0) at 20 °C and 30 °C. Values represent the mean ± SD of three independent experiments (n = 3).

		**Alkene (%)**	**β-Hydroxylation (%)**	**α-Hydroxylation (%)**	**Decarboxylation/ Hydroxylation Ratio**
**Activity at 20 ºC**	C10:0	78.4 ± 1.2	16.2 ± 1.6	-	4.8
	C12:0	79.9 ± 1.8	18.0 ± 2.0	-	4.4
	C14:0	81.5 ± 1.1	15.6 ± 1.0	-	5.2
	C16:0	58.8 ± 1.6	9.5 ± 0.6	-	6.2
	C18:0	23.6 ± 1.0	-	-	-
	C20:0	3.4 ± 2.4	-	-	-
**Activity at 30 ºC**	C10:0	64.0 ± 1.8	21.0 ± 0.1	1.4 ± 0.2	2.9
	C12:0	68.3 ± 0.5	28.6 ± 0.2	1.8 ± 0.1	2.2
	C14:0	67.8 ± 11.8	21.8 ± 2.2	1.7 ± 0.1	2.9
	C16:0	77.0 ± 6.9	12.6 ± 5.9	0.9 ± 0.5	5.7
	C18:0	61.9 ± 2.1	4.5 ± 0.3	1.5 ± 0.1	10.3
	C20:0	11.6 ± 3.4	-	-	-

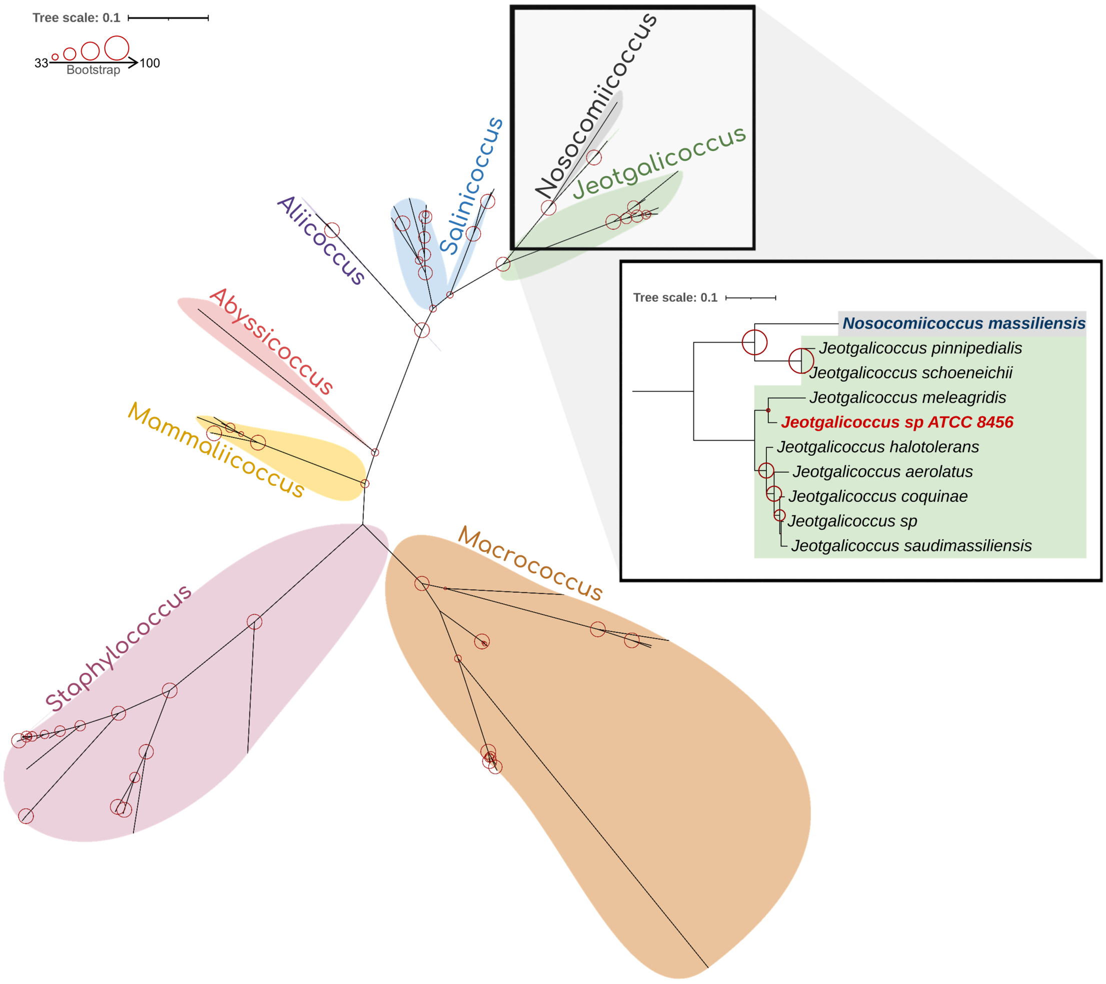

Figure S1. Phylogenetic positioning of $OleT_{JE}$ and $OleT_{NS}$ sequences. An unrooted maximum-likelihood tree comprising 60 sequences from the $OleT_{JE}$ cluster is displayed, with different genera colour-coded: *Macrococcus* (light tan), *Staphylococcus* (pale pink), *Mammaliicoccus* (yellow), *Abyssicoccus* (light coral), *Aliicoccus* (lavender), *Salinicoccus* (light blue), *Nosocomiicoccus* (gray), and *Jeotgalicoccus* (light green). The inset presents a structure-based reconstructed tree of the $OleT_{JE}$/$OleT_{NS}$ subclade, including *Jeotgalicoccus* sp. ATCC 8456 ($OleT_{JE}$) and *Nosocomiicoccus massiliensis* ($OleT_{NS}$). Bootstrap support is indicated by red circles, and branch-length scales are shown for both the main tree and the inset.

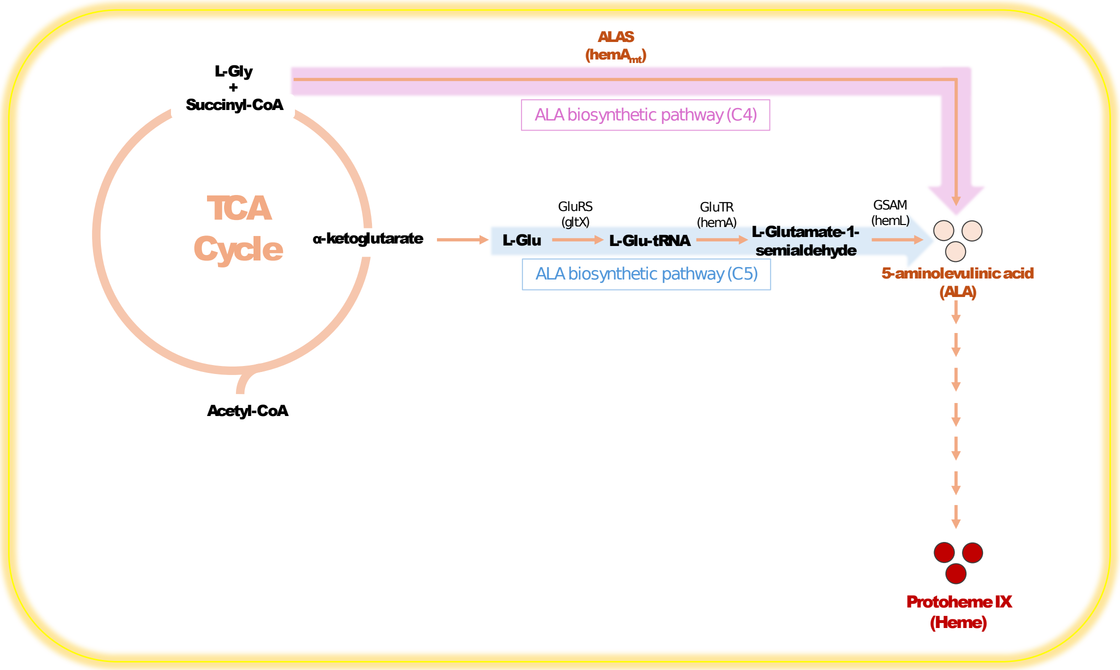

Figure S2. Schematic representation of the optimized heme biosynthesis pathway in *Escherichia coli*. The endogenous C5 pathway, which converts glutamate to 5-aminolevulinic acid (ALA), remains active, while the heterologous C4 pathway, enabled via expression of hemA$_{mt}$, converts glycine and succinyl-CoA into ALA. The combined activity of both pathways increases intracellular heme levels, enhancing the expression of functional P450 enzymes. Abbreviations: L-Gly, glycine; L-Glu, glutamate; L-Glu-tRNA, glutamyl-tRNA.

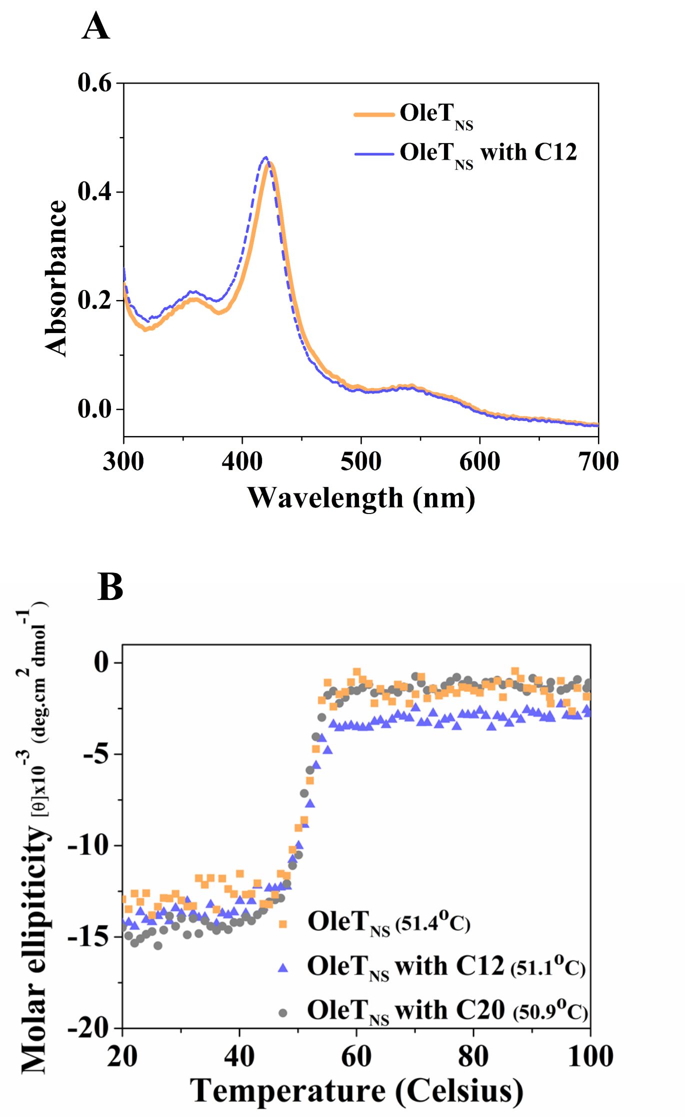

Figure S3. (**A**) UV-visible spectra of purified substrate-free $OleT_{NS}$ (5 µM, orange solid line) and $OleT_{NS}$ bound to dodecanoic acid (150 µM, purple dashed line). (**B**) Thermal unfolding profiles measured by ellipticity at 222 nm of $OleT_{NS}$ alone (orange), $OleT_{NS}$ in complex with dodecanoic acid (purple), and $OleT_{NS}$ in complex with arachidonic acid (gray).

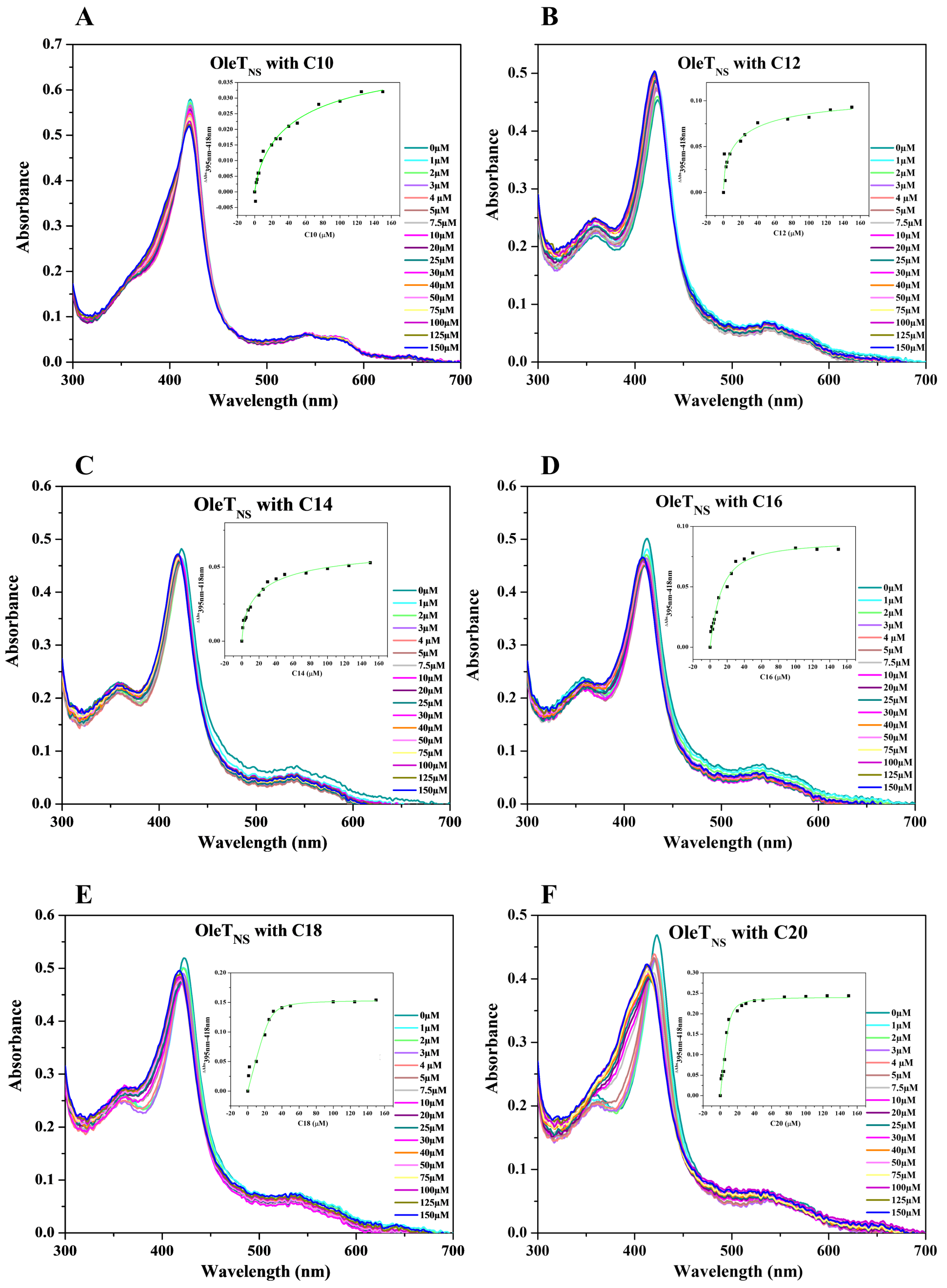

Figure S4. Substrate binding to OleT$_{NS}$ enzyme (5 μM), showing the transition from Low-Spin (~418 nm) to High-Spin (~396 nm). Spectral titrations and corresponding Kd values are shown for: (A) decanoic acid (C10), (B) lauric acid (C12), (C) myristic acid (C14), (D) palmitic acid (C16), (E) stearic acid (C18), and (F) arachidonic acid (C20). Binding data were fitted using the Morrison equation, providing the *K*d values reported in Table S4.

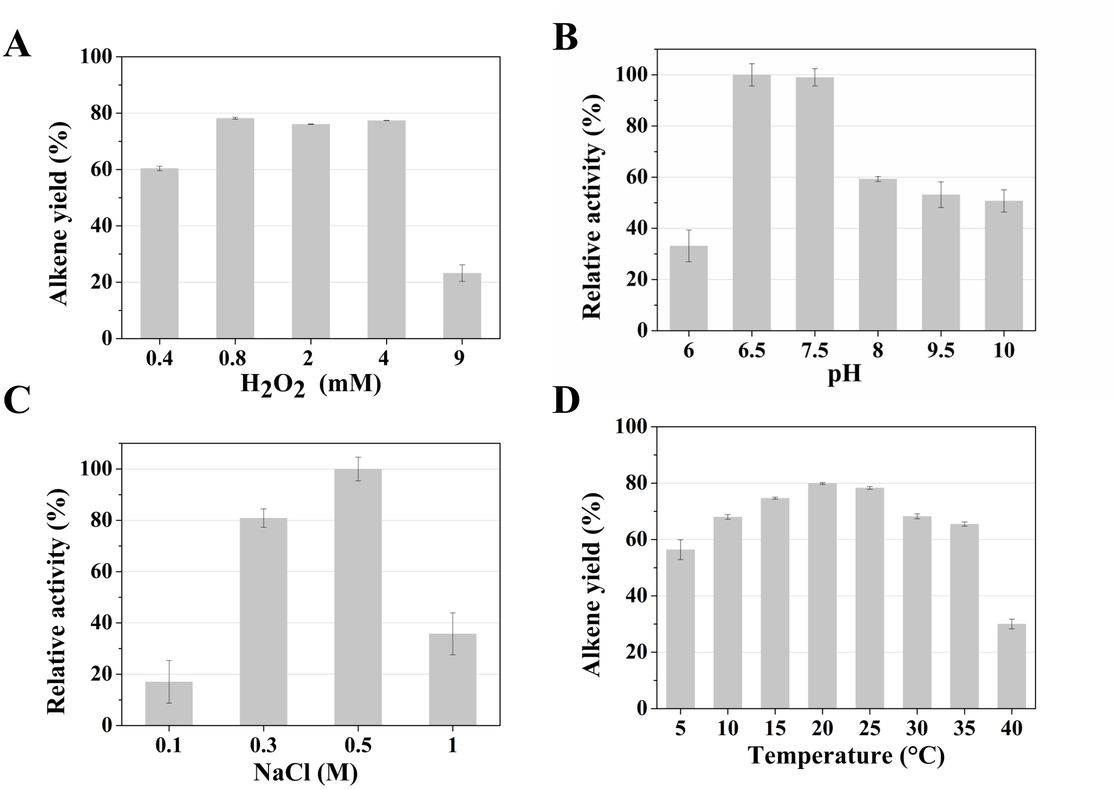

Figure S5. Biochemical characterization of $OleT_{NS}$. **(A)** Relative activity across varying hydrogen peroxide concentrations. **(B)** pH-dependent activity profile. **(C)** Effect of salt concentration. **(D)** Temperature-dependent activity profile. Data are shown as mean ± SD from three independent experiments (n = 3), with activities expressed as relative activity (%).

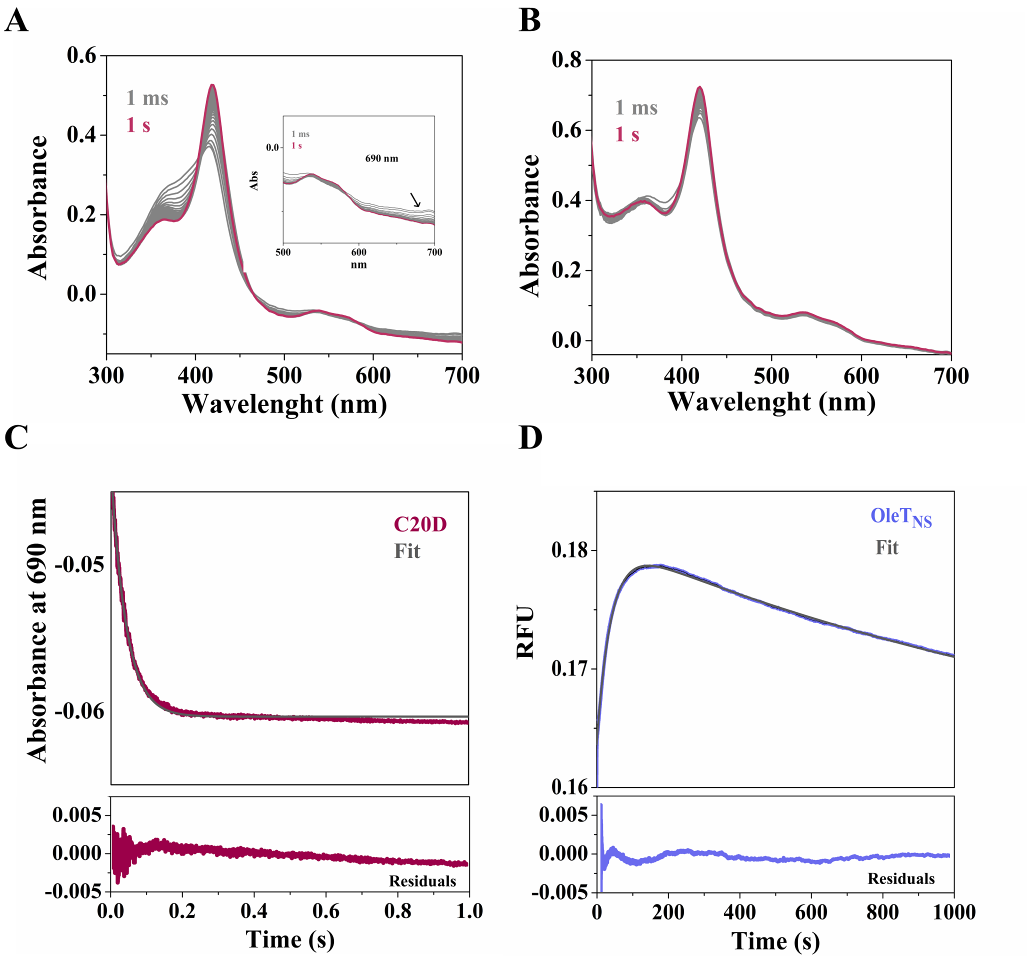

Figure S6. Photodiode array spectra showing the rapid decay of Compound I and the subsequent formation and decay of Compound II upon reaction of OleT$_{NS}$ with **(A)** deuterated (C20D) or **(B)** protonated (C20H) arachidonic acid in the presence of excess H_2O_2 at 4 °C. Final concentrations after mixing were 10 µM OleT$_{NS}$ and 5 mM H_2O_2. Insets highlight changes in the Soret region. Single-wavelength kinetic traces fitted to exponential models. **(C)** Absorbance at 690 nm for C20D, showing the decay of Compound I; residuals are plotted below. **(D)** Transient fluorescence changes of the OleT$_{NS}$:DAUDA ternary complex upon rapid mixing with excess H_2O_2 at 4 °C. Concentrations were 10 µM OleT$_{NS}$, 20 µM DAUDA (2 equivalents), and 2 mM H_2O_2. Fluorescence was monitored using a PMT detector (excitation 280 nm); fitted traces and residuals are shown.

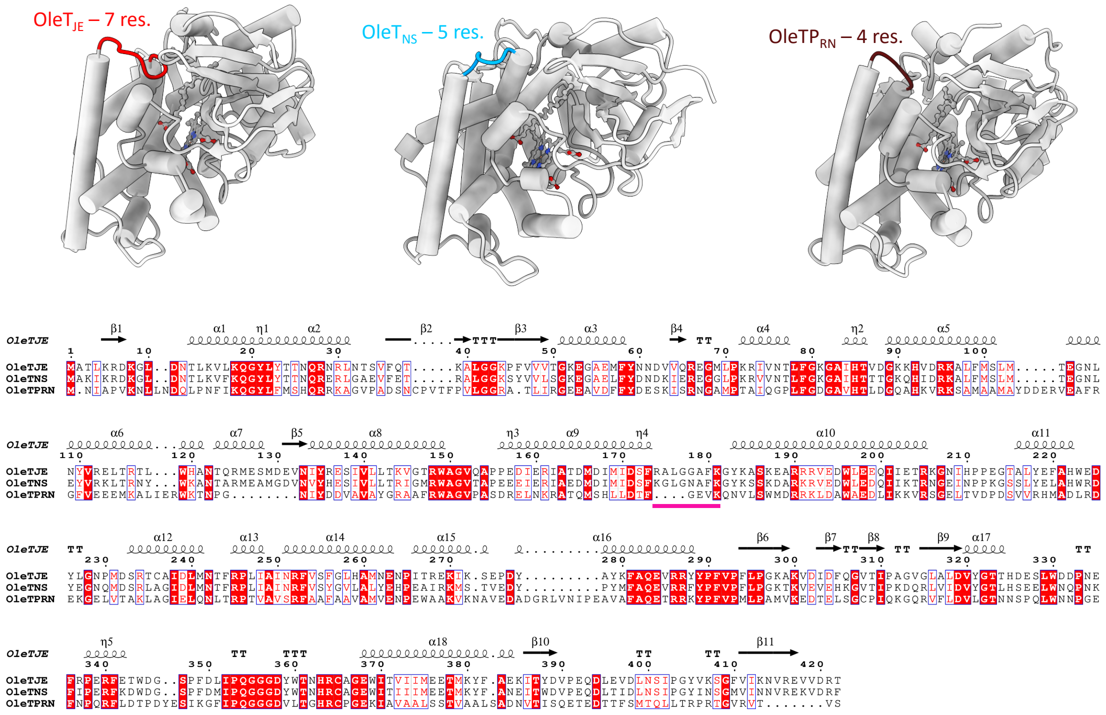

Figure S7. Structural predictions and sequence alignment of the F-G loop in OleT enzymes. The AlphaFold3-predicted structure of OleT$_{NS}$ is shown with its F-G loop highlighted in cyan, in comparison with the corresponding regions of OleT$_{JE}$ (red; PDB: 4L40) and OleTP$_{RN}$ (brown; PDB: 8D8P). The number of residues in each F-G loop is indicated for each enzyme. The sequence alignment of OleT enzymes is displayed below the structures, with secondary structure elements from OleT$_{JE}$ indicated above each row. The F-G loop region is highlighted in pink, allowing direct comparison of loop length and composition across the three enzymes.

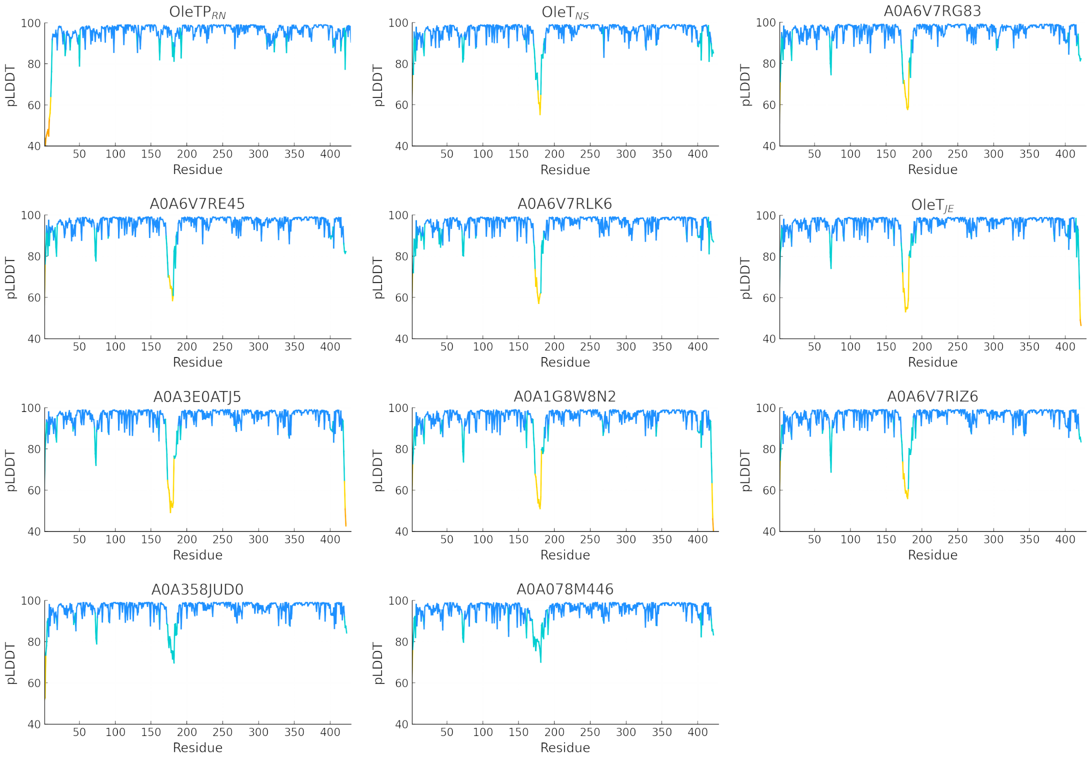

Figure S8. Per-residue confidence of AlphaFold3 models. Predicted local distance difference test (pLDDT) scores are shown for the top-ranked structural models used in this study. Confidence levels are color-coded: very high (> 90, blue), high (70-90, cyan), low (50-70, yellow), and very low (< 50, orange). F-G loop regions are indicated. Enzyme names or UniProt codes are listed for the following organisms: *Rothia nasimurium* (OleTP$_{RN}$, A0A1Y1RQ53), *Nosocomiicoccus massiliensis* (OleT$_{NS}$, A0A2J6NJM5), *Phocicoccus pinnipedialis* (A0A6V7RG83), *Phocicoccus schoeneichii* (A0A6V7RE45), *Jeotgalicoccus meleagridis* (A0A6V7RLK6), *Jeotgalicoccus* sp. ATCC 8456 (OleT$_{JE}$, E9NSU2), *Jeotgalicoccus halotolerans* (A0A3E0ATJ5), *Jeotgalicoccus aerolatus* (A0A1G8W8N2), *Jeotgalicoccus coquinae* (A0A6V7RIZ6), *Jeotgalicoccus* sp. (A0A358JUD0), and *Jeotgalicoccus saudimassiliensis* (A0A078M446).

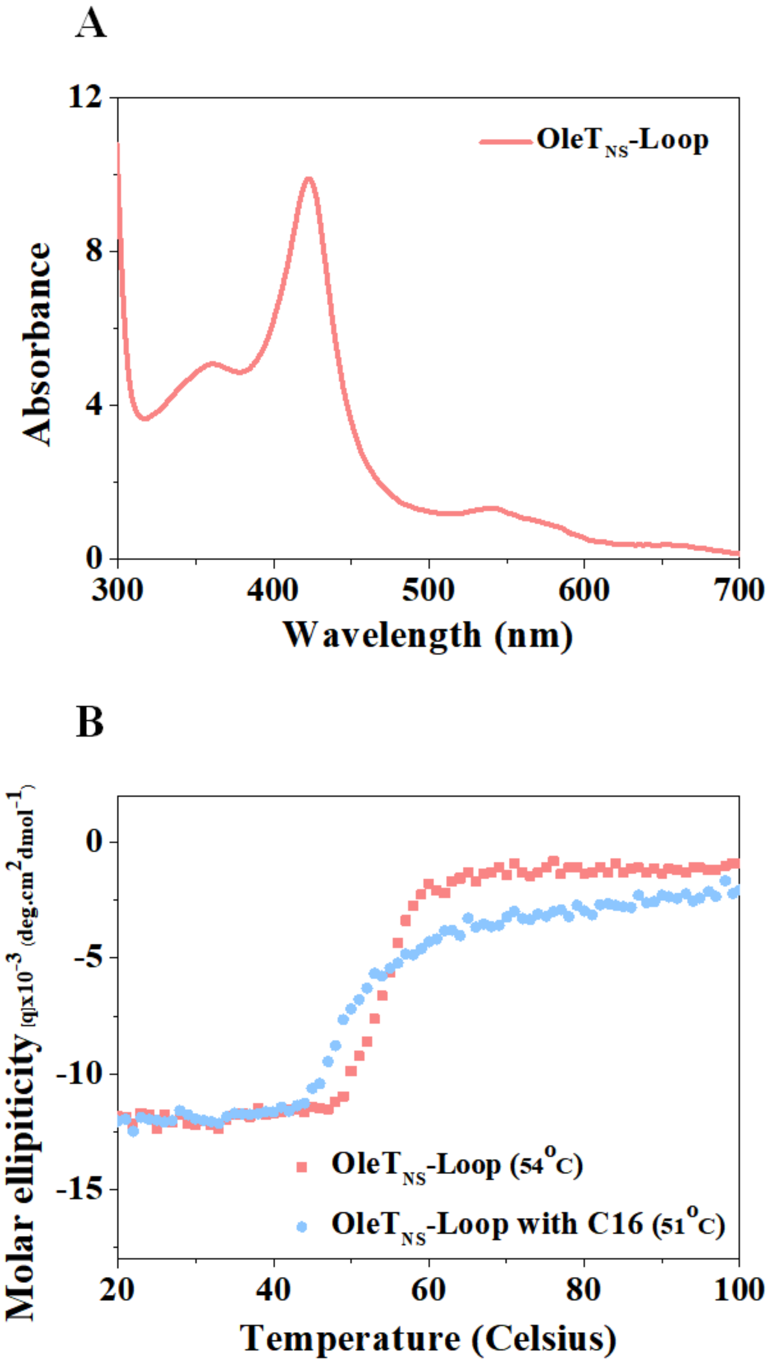

Figure S9. Biophysical and functional characterization of $OleT_{NS}$-Loop. **(A)** UV-visible absorption spectra of purified substrate-free $OleT_{NS}$-Loop, highlighting characteristic heme signatures. **(B)** Thermal denaturation monitored by circular dichroism at 222 nm, comparing $OleT_{NS}$-Loop alone (pink) with $OleT_{NS}$-Loop bound to stearic acid (blue), illustrating stabilization upon substrate binding.

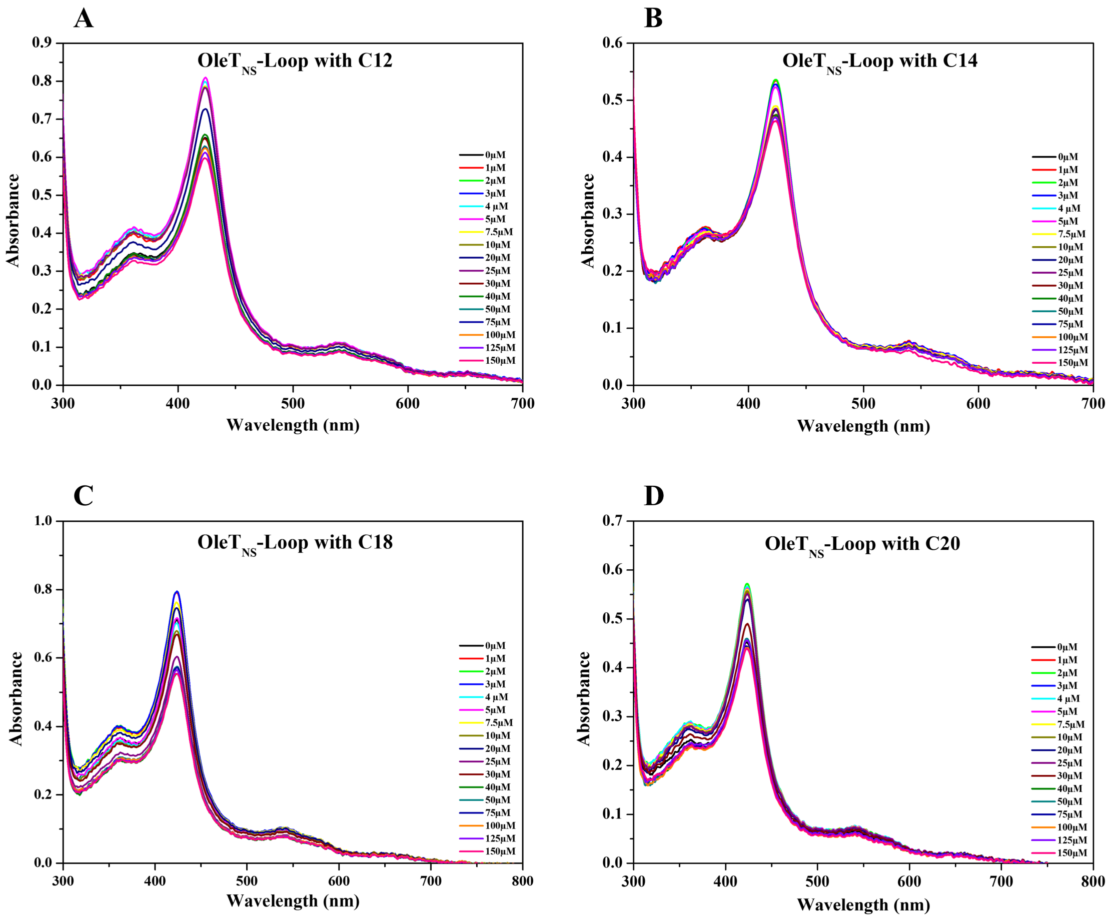

Figure S10. Substrate titration of the OleT$_{NS}$-Loop enzyme (5 μM) showed no detectable transition from the low-spin state to the high-spin state. Spectral titrations were performed with (A) lauric acid (C12), (B) myristic acid (C14), (C) stearic acid (C18), and (D) arachidonic acid (C20).

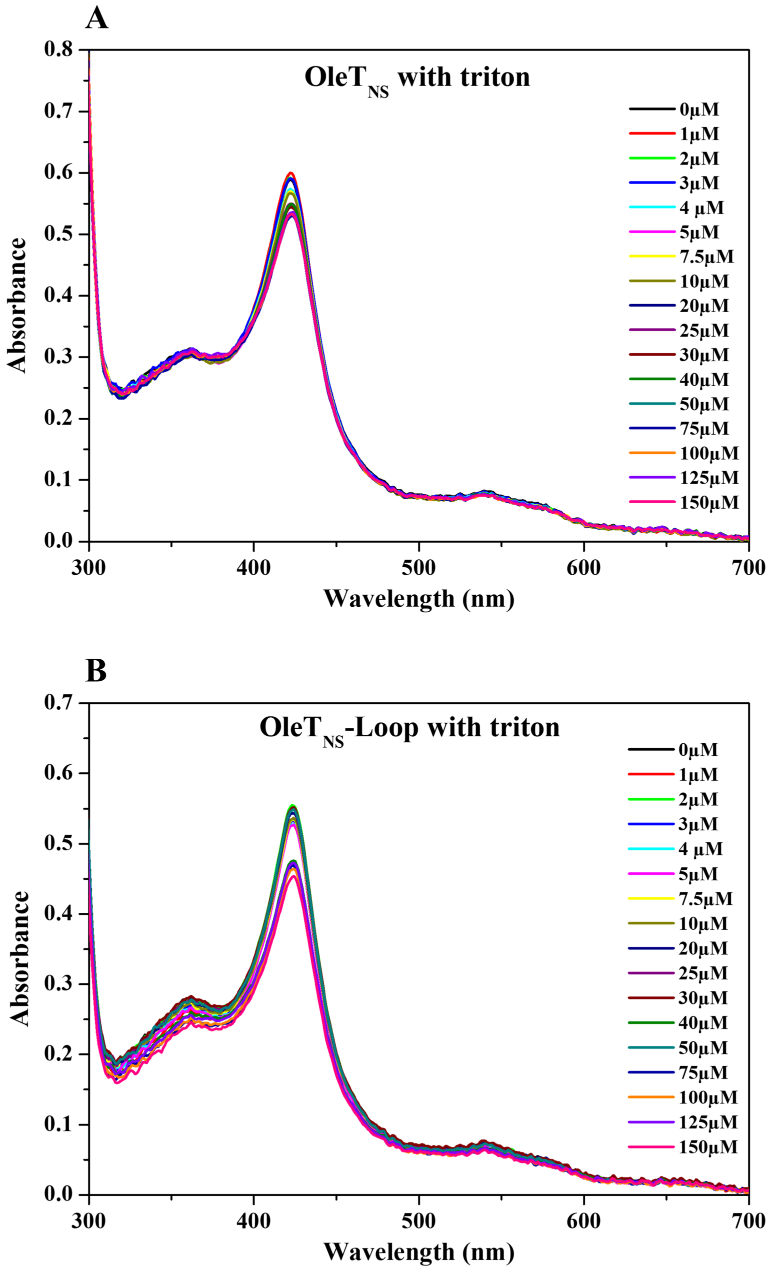

Figure S11. Titration of Triton X-100 (30% in ethanol) into OleT$_{NS}$ (A) and OleT$_{NS}$-Loop (B) (5 µM). No detectable binding was observed for either enzyme.

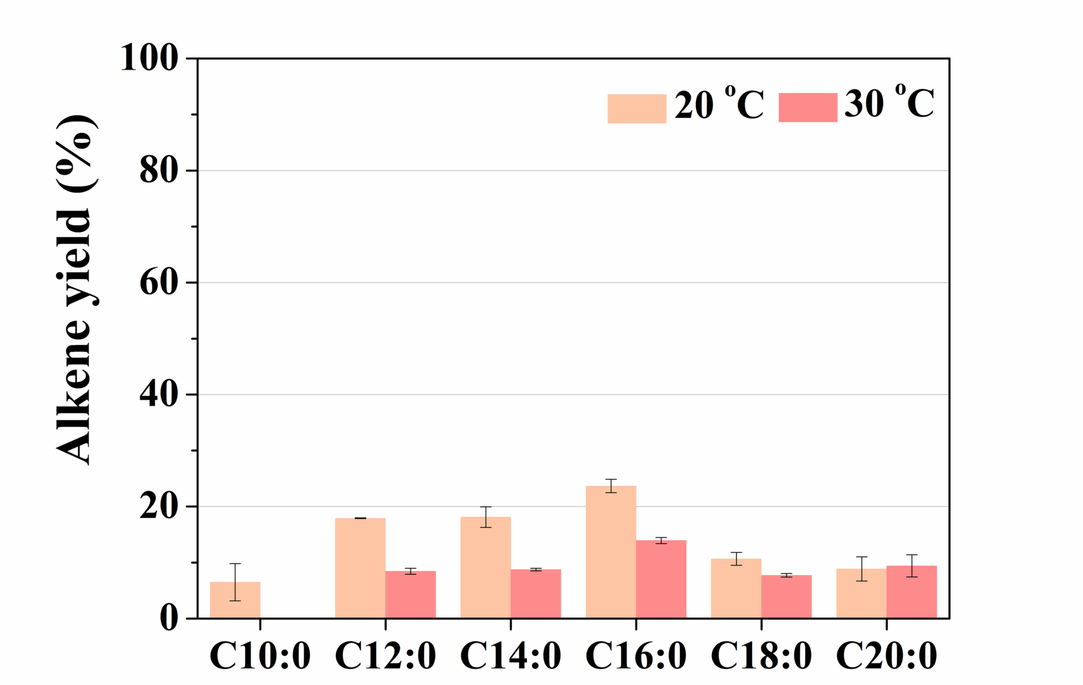

Figure S12. Catalytic activity of OleT$_{NS}$-Loop toward saturated fatty acids (C10:0-C20:0) at 20 °C (light orange) and 30 °C (light pink), expressed as alkene yield (%).

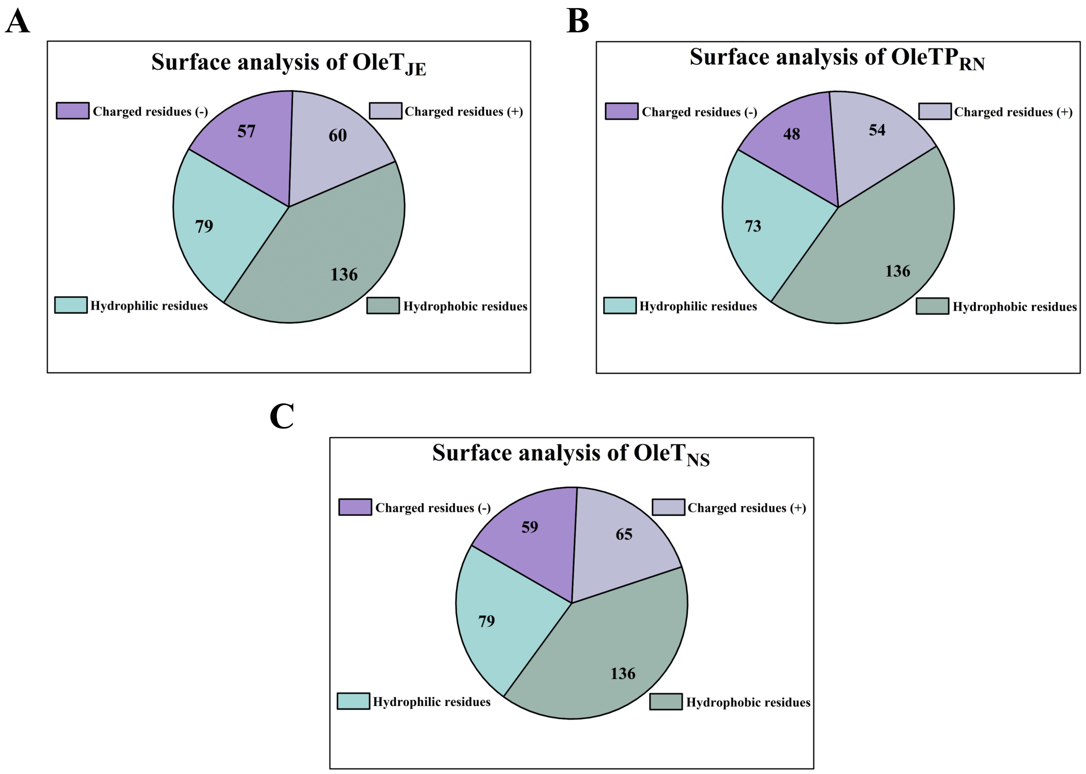

Figure S13. Surface property analysis of peroxygenases **(A)** OleT$_{JE}$, **(B)** OleTP$_{RN}$, and **(C)** OleT$_{NS}$. PyMOL was used to map surface residues according to physicochemical characteristics: negatively charged residues (dark purple), positively charged residues (light purple), hydrophilic residues (light green), and hydrophobic residues (dark green). OleT$_{NS}$ exhibits the highest density of surface-exposed charged residues, suggesting distinctive electrostatic properties compared with OleT$_{JE}$ and OleTP$_{RN}$.

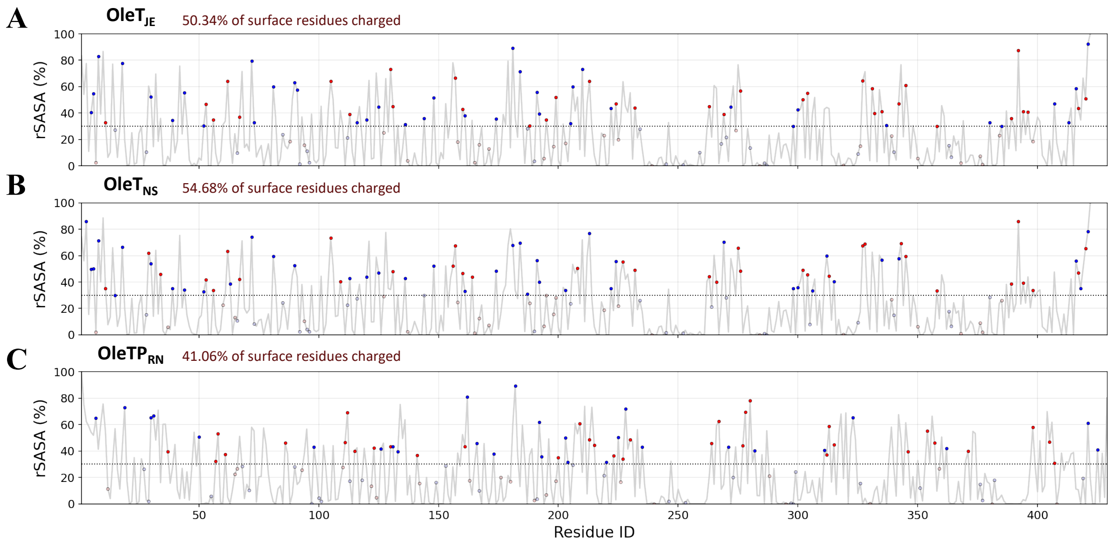

Figure S14. Solvent accessibility and surface charge distribution of OleT enzymes. Relative solvent-accessible surface area (rSASA) was calculated for each residue of **(A)** OleT$_{JE}$, **(B)** OleT$_{NS}$, and **(C)** OleTP$_{RN}$. Residues with rSASA > 30% were classified as surface-exposed. Among these, positively charged residues are highlighted in blue and negatively charged residues in red, while buried residues are shown with reduced color saturation. The percentage of charged residues among all surface-exposed residues is indicated for each enzyme, revealing distinct electrostatic profiles that may influence substrate interaction and enzyme stability.

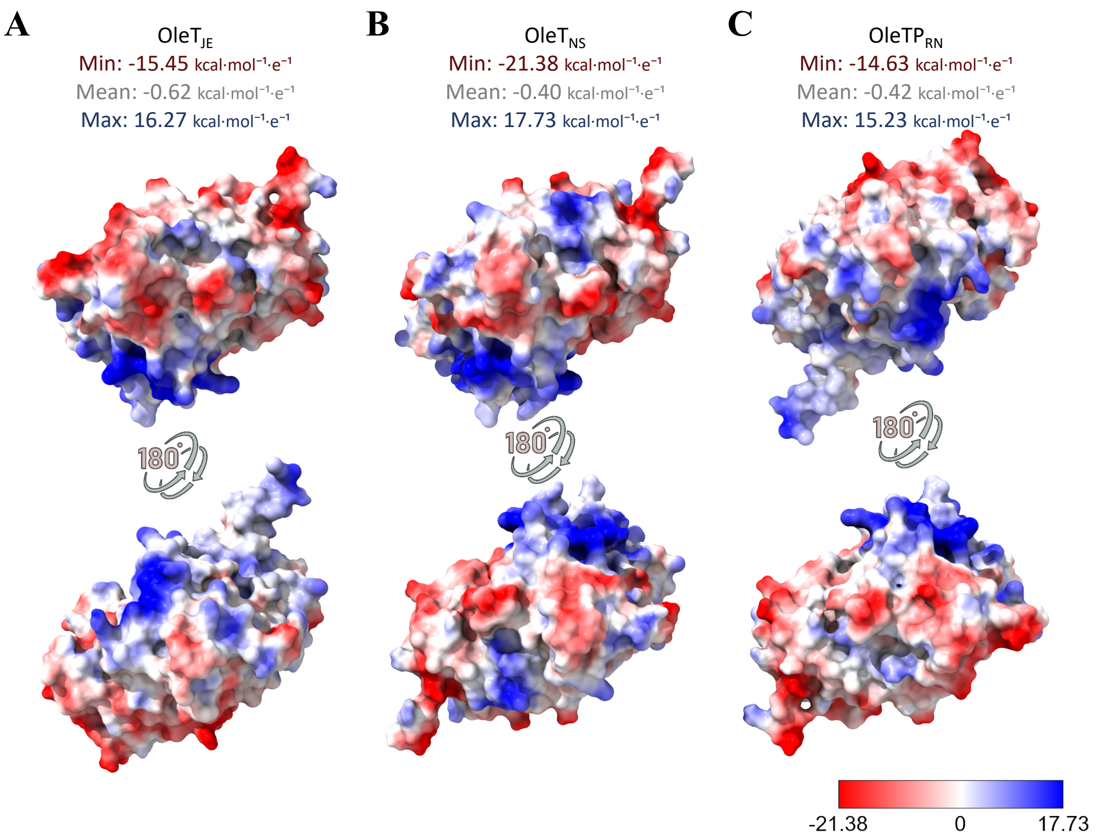

Figure S15. Comparative electrostatic surface potentials of OleT enzymes. Surface electrostatic potentials of **(A)** OleT$_{JE}$, **(B)** OleT$_{NS}$, and **(C)** OleTP$_{RN}$ were computed using ChimeraX. Each protein is presented in two orthogonal views (rotated 180°) to highlight global charge distributions. Potentials are mapped from negative (red) to positive (blue) values, with the scale in kcal·mol^{-1}·e^{-1}. Minimum, mean, and maximum potential values are provided for each enzyme to facilitate comparison of surface charge heterogeneity and potential interaction sites relevant for substrate binding and catalytic activity.

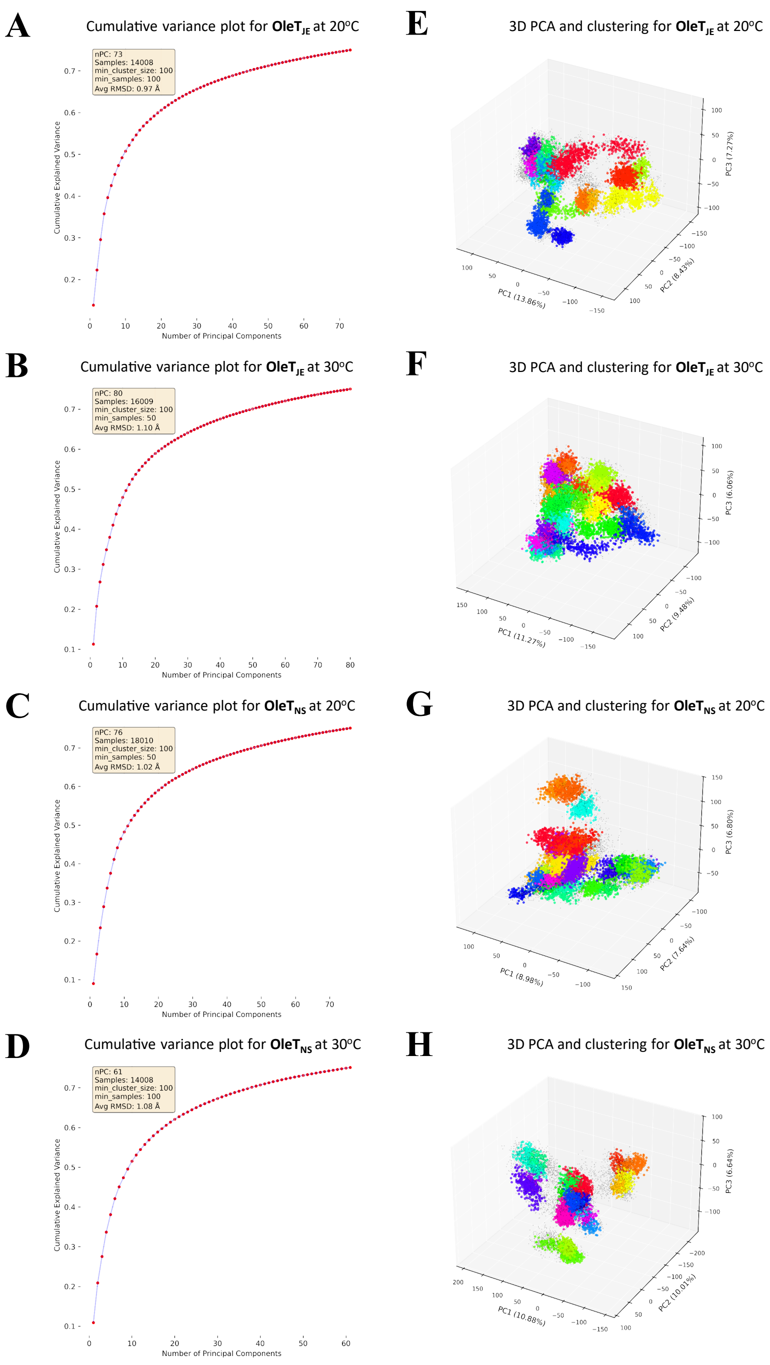

Figure S16. Principal component analysis (PCA) of $OleT_{JE}$ and $OleT_{NS}$ conformational dynamics. Cumulative variance captured by principal components is presented alongside clustering metrics for $OleT_{JE}$ at 20 °C **(A)** and 30 °C **(B)**, and for $OleT_{NS}$ at 20 °C **(C)** and 30 °C **(D)**. Corresponding conformational projections along the first three principal components are shown, with points colored according to cluster assignment, for $OleT_{JE}$ at 20 °C **(E)** and 30 °C **(F)**, and for $OleT_{NS}$ at 20 °C **(G)** and 30 °C **(H)**. Clustering statistics reported include the number of principal components retained, total number of sampled conformations, minimum cluster size, and the average root-mean-square deviation (RMSD) within clusters, providing a quantitative assessment of structural heterogeneity.

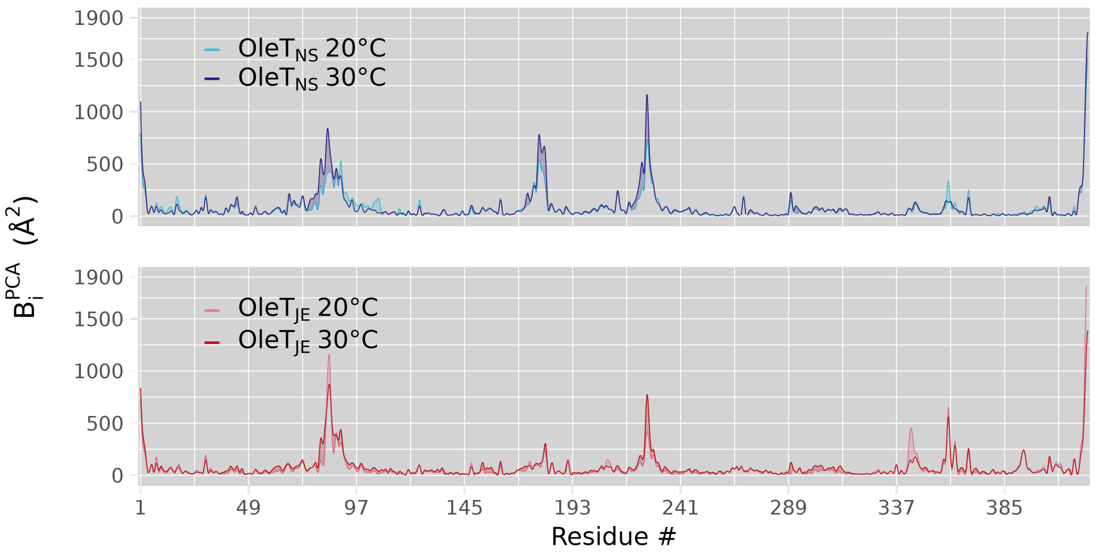

Figure S17. Temperature-dependent essential dynamics profiles of OleT enzymes. (A) OleT$_{NS}$ (blue) and (B) OleT$_{JE}$ (red) show residue-wise PCA-derived temperature factors, BiPCA in Å2. Lighter shades correspond to simulations at 20 °C and darker shades to 30 °C. Only protein residues are shown.

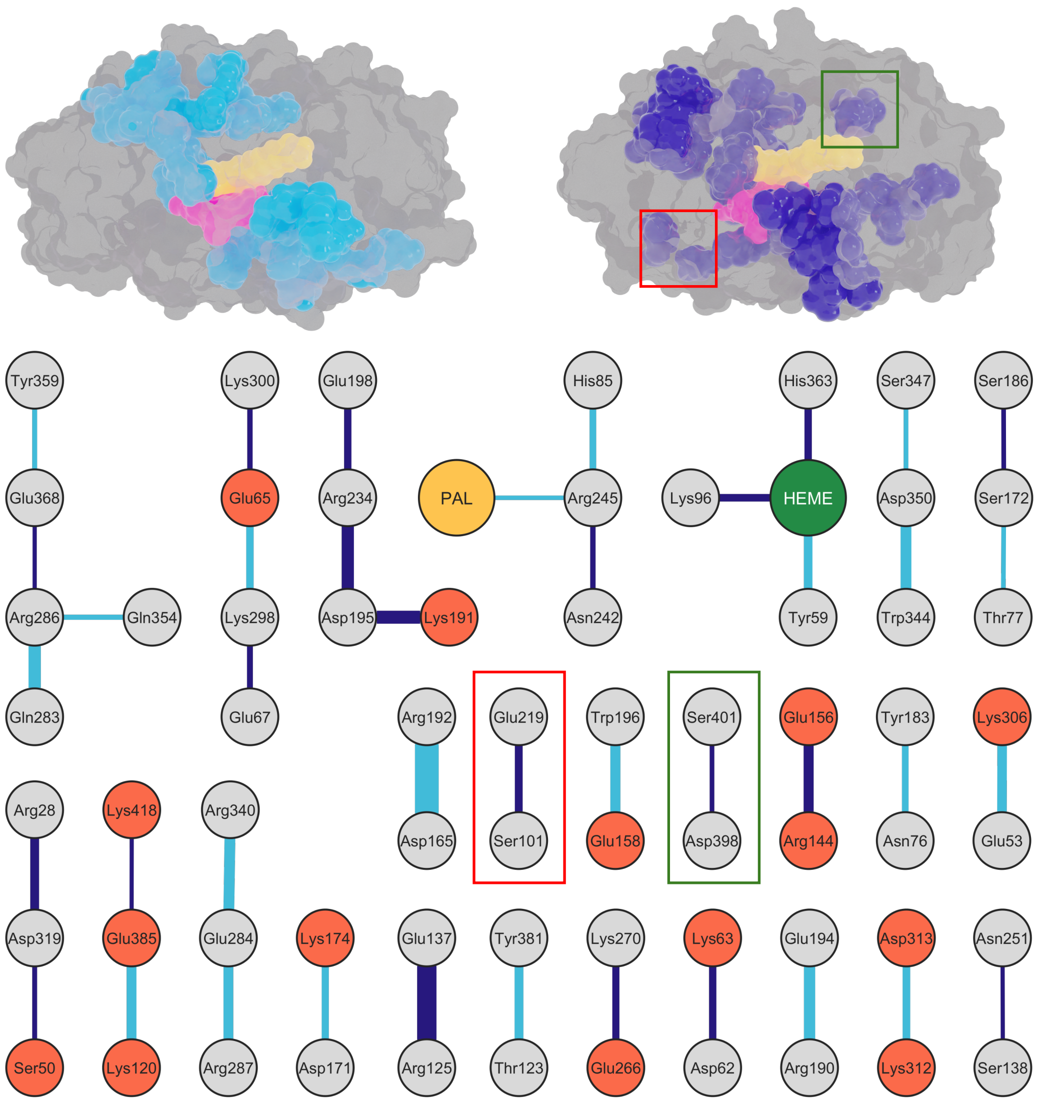

Figure S18. Temperature-dependent hydrogen bond dynamics in $\mathrm{OleT_{NS}}$ from PCA-derived trajectories. The solvent-accessible surface of $\mathrm{OleT_{NS}}$ is depicted (top), with interacting atoms colored according to hydrogen bond frequency differences between simulations at 20 °C and 30 °C. Interactions preferentially formed at 20 °C are highlighted in light blue, while those enriched at 30 °C are shown in dark blue. Below, a network representation of residue-residue interactions illustrates these differential contacts, with edge thickness proportional to the relative change in hydrogen bond frequency between temperatures. Residue nodes in red correspond to positions that are non-conserved in $\mathrm{OleT_{JE}}$. The heme cofactor (HEME) is indicated as a green node, and palmitate (PAL) is represented as a yellow node, enabling visualization of temperature-dependent modulation of the active site and neighboring interactions.

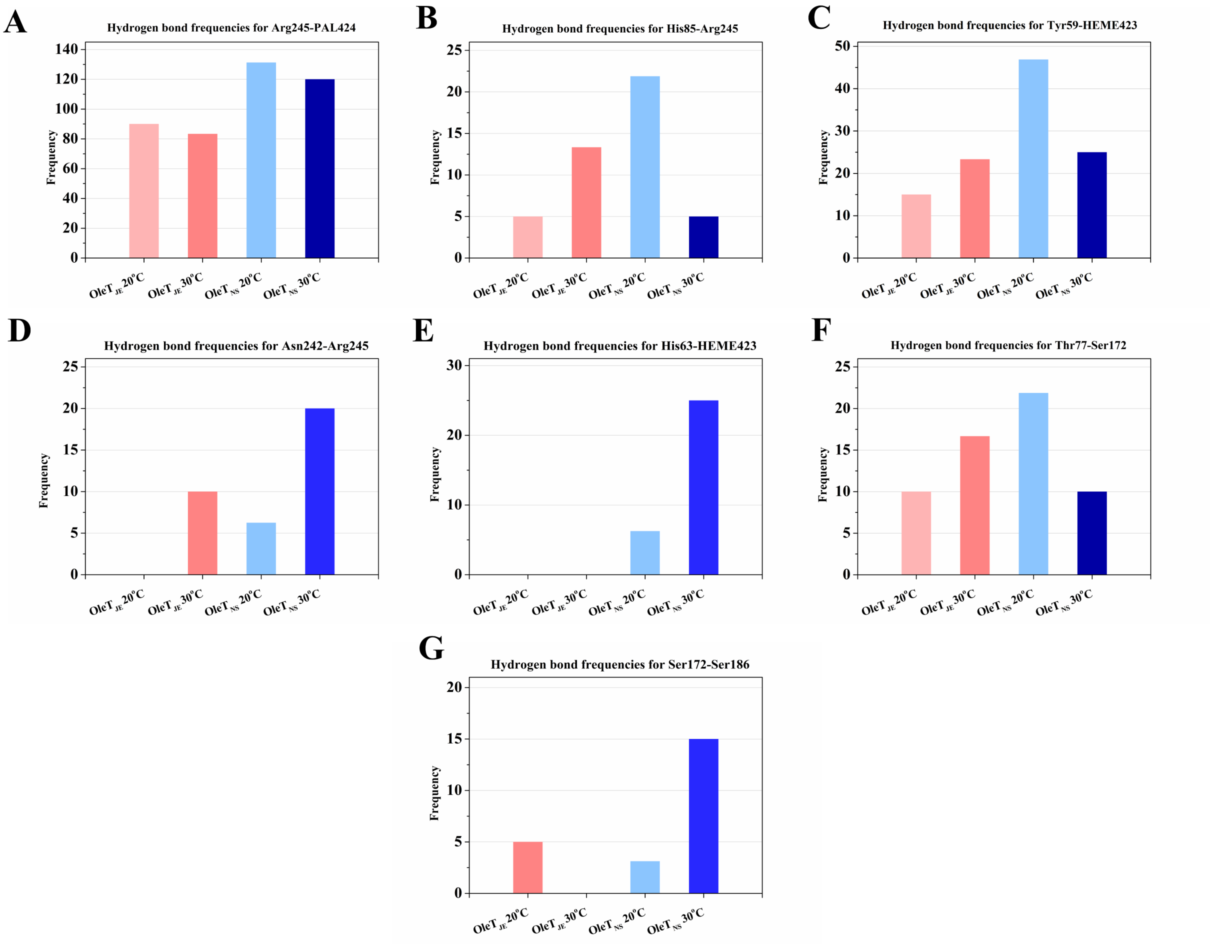

Figure S19 Hydrogen bond frequencies were analyzed between **(A)** Arg245-Palmitic acid, **(B)** His25-Arg245, **(C)** Thr59-HEME423, **(D)** Asn242-Arg245, **(E)** His63-HEME423, **(F)** Thr77-Ser172, and **(G)** Ser172-Ser186. Molecular dynamics simulations were performed to investigate the influence of temperature on $OleT_{NS}$ chemoselectivity. Structural regions involved in catalysis, including the heme active site, substrate, F-G loop, and key catalytic residues, were evaluated for hydrogen bond frequencies at 20 °C and 30 °C. For comparison, the same analysis was conducted on $OleT_{JE}$, a well-characterized decarboxylase from *Jeotgalicoccus*. Results showed that $OleT_{NS}$ exhibited higher hydrogen bond frequencies at 20 °C for those catalytic regions, along with a significant conformational change in the F-G loop that pushes the substrate toward the catalytic pocket, potentially favoring β-carbon activity.

Movie S1. $OleT_{NS}$ Catalytic Mechanism with palmitic acid (C16:0, yellow) at 20 °C shows increased hydrogen bond frequencies between residue pairs Arg245–His85 and Ser172–Thr77 (light blue), which help stabilize the substrate in the catalytic pocket by keeping it in a closed conformation, aided by the F-G loop movement that pushes the substrate toward the active site. At 30 °C, residue pairs Arg245–Asn242 and Ser172–Ser186 (dark blue) form stronger interactions, resulting in a more open catalytic pocket and less conformational constraint for substrate positioning. The heme B group is shown in hot pink.

REFERENCES

1. Micsonai, A., Wien, F., BulyákiBuly, E., Kun, J., Moussong, E., Lee, Y.-H., Goto, Y., and Kardos, ozsef (2018) BeStSel: a web server for accurate protein secondary structure prediction and fold recognition from the circular dichroism spectra. *Nucleic Acids Res.* **46**, 315–322

2. Rade, L. L., Generoso, W. C., Das, S., Souza, A. S., Silveira, R. L., Avila, M. C., Vieira, P. S., Miyamoto, R. Y., Lima, A. B. B., Aricetti, J. A., de Melo, R. R., Milan, N., Persinoti, G. F., Bonomi, A. M. F. L. J., Murakami, M. T., Makris, T. M., and Zanphorlin, L. M. (2023) Dimer-assisted mechanism of (un)saturated fatty acid decarboxylation for alkene production. *Proc. Natl. Acad. Sci. U. S. A.* **120**, e2221483120
